# Human pancreatic cancer cells under nutrient deprivation are vulnerable to redox system inhibition

**DOI:** 10.1074/jbc.RA120.013893

**Published:** 2021-01-13

**Authors:** Takefumi Onodera, Isao Momose, Hayamitsu Adachi, Yohko Yamazaki, Ryuichi Sawa, Shun-ichi Ohba, Manabu Kawada

**Affiliations:** 1 Institute of Microbial Chemistry (BIKAKEN), Numazu, Shizuoka, Japan; 2 Institute of Microbial Chemistry (BIKAKEN), Tokyo, Japan

**Keywords:** redox regulation, oxidation reduction (redox), oxidative stress, thioredoxin, metabolism, penicillic acid, papyracillic acid, auranofin, glutathione, chemical biology, drug discovery, drug screening, cancer therapy

## Abstract

Large regions in tumor tissues, particularly pancreatic cancer, are hypoxic and nutrient-deprived because of unregulated cell growth and insufficient vascular supply. Certain cancer cells, such as those inside a tumor, can tolerate these severe conditions and survive for prolonged periods. We hypothesized that small molecular agents, which can preferentially reduce cancer cell survival under nutrient-deprived conditions, could function as anticancer drugs. In this study, we constructed a high-throughput screening system to identify such small molecules and screened chemical libraries and microbial culture extracts. We were able to determine that some small molecular compounds, such as penicillic acid, papyracillic acid, and auranofin, exhibit preferential cytotoxicity to human pancreatic cancer cells under nutrient-deprived compared with nutrient-sufficient conditions. Further analysis revealed that these compounds target to redox systems such as GSH and thioredoxin and induce accumulation of reactive oxygen species in nutrient-deprived cancer cells, potentially contributing to apoptosis under nutrient-deprived conditions. Nutrient-deficient cancer cells are often deficient in GSH; thus, they are susceptible to redox system inhibitors. Targeting redox systems might be an attractive therapeutic strategy under nutrient-deprived conditions of the tumor microenvironment.

Pancreatic cancer is an aggressive disease that frequently presents at an advanced stage (1), and the majority of the patients with pancreatic cancer have surgically unresectable disease. Pancreatic cancer is a major cause of cancer-associated mortality, and the 5-year survival rate is only 8% (2). Effective drug therapies for patients with pancreatic cancer are desired.

The tumor microenvironment plays an important role in tumor progression and metastasis; thus this should be a potential target for anticancer drugs (3, 4). Large regions of tumor tissues are often nutrient-deprived and hypoxic because of aberrant cell proliferation and abnormal blood vessels (5, 6). In particular, pancreatic cancer tumors are typically hypoxic and low in nutrients because of poor vascularization and high interstitial pressure (7, 8, 9, 10). Certain cancer cells can tolerate severe conditions, such as poor nutrient availability and oxygen deprivation and survive for prolonged periods (11). To survive in a nutrient-deprived environment, cancer cells use the phosphoinositide 3-kinase/Akt pathway that is associated with nutrient acquisition, cell proliferation, and apoptosis inhibition (12, 13). Previously, we showed that kigamicins (polycyclic xanthone compounds produced by *Amycolatopsis* sp. ML630-mF1) inhibit Akt activation and demonstrate preferential cytotoxicity to human pancreatic cancer cells under nutrient-deprived conditions compared with nutrient-sufficient conditions (14, 15, 16). Therefore, targeting cancer cells that have adapted to nutrient deprivation might be an effective strategy for cancer therapy.

Further, the tumor microenvironment plays a major role in determining the metabolic phenotypes of cancer cells (17). Metabolic alterations affect reactive oxygen species (ROS) production, which modulates the cellular reduction–oxidation (redox) status (18). Pancreatic cancer is characterized by the expression of oncogenic KRAS, and >90% of the patients with pancreatic cancer have oncogenic mutations in the *KRAS* (19). Oncogenic KRAS induces ROS production through activation of NADPH oxidase 1 and mitochondrial dysfunction (20, 21). KRAS-induced ROS causes DNA damage and genomic instability, which, in turn, facilitates the acquisition of malignant phenotypes (21). Appropriate levels of ROS are deemed beneficial for tumor development and progression; however, an excess of ROS leads to senescence and cell death because ROS can damage DNA, RNA, lipids, and proteins.

The cells use redox systems, such as GSH and thioredoxin (Trx) systems, to counteract the detrimental effects of ROS. The GSH system involves NADPH, GSH reductase, GSH, and GSH peroxidase, whereas the Trx systems involves NADPH, thioredoxin reductase (TrxR), Trx, and peroxiredoxin (22). GSH peroxidase and peroxiredoxin are antioxidant enzymes that efficiently catalyze the decomposition of H_2_O_2_ and that are known to reduce excessive ROS levels and prevent cellular damage. Many genes involved in the GSH and Trx systems are regulated by the transcription factor Nrf2 (nuclear factor erythroid 2–related factor 2). Oncogenic KRAS induces Nrf2 transcription and promotes ROS detoxification that supports pancreatic tumor maintenance (23, 24).

Cancer cells exhibit often high ROS levels compared with normal cells because of metabolic and signaling aberrations caused by the accumulation of multiple genetic alterations (25, 26). Accordingly, these malignant cells are more dependent on antioxidants for cell survival and thus are more vulnerable to further oxidative stress induced by inhibition of redox systems (26, 27). In the present study, we found that inhibitors of redox system display preferential cytotoxicity to cancer cells in nutrient-deprived conditions. Targeting redox systems might be an attractive therapeutic strategy under nutrient-deprived conditions of the tumor microenvironment.

## Results

### PCA and PPA preferentially inhibit human pancreatic cancer cell growth under nutrient-deprived conditions

**Figure 1:**
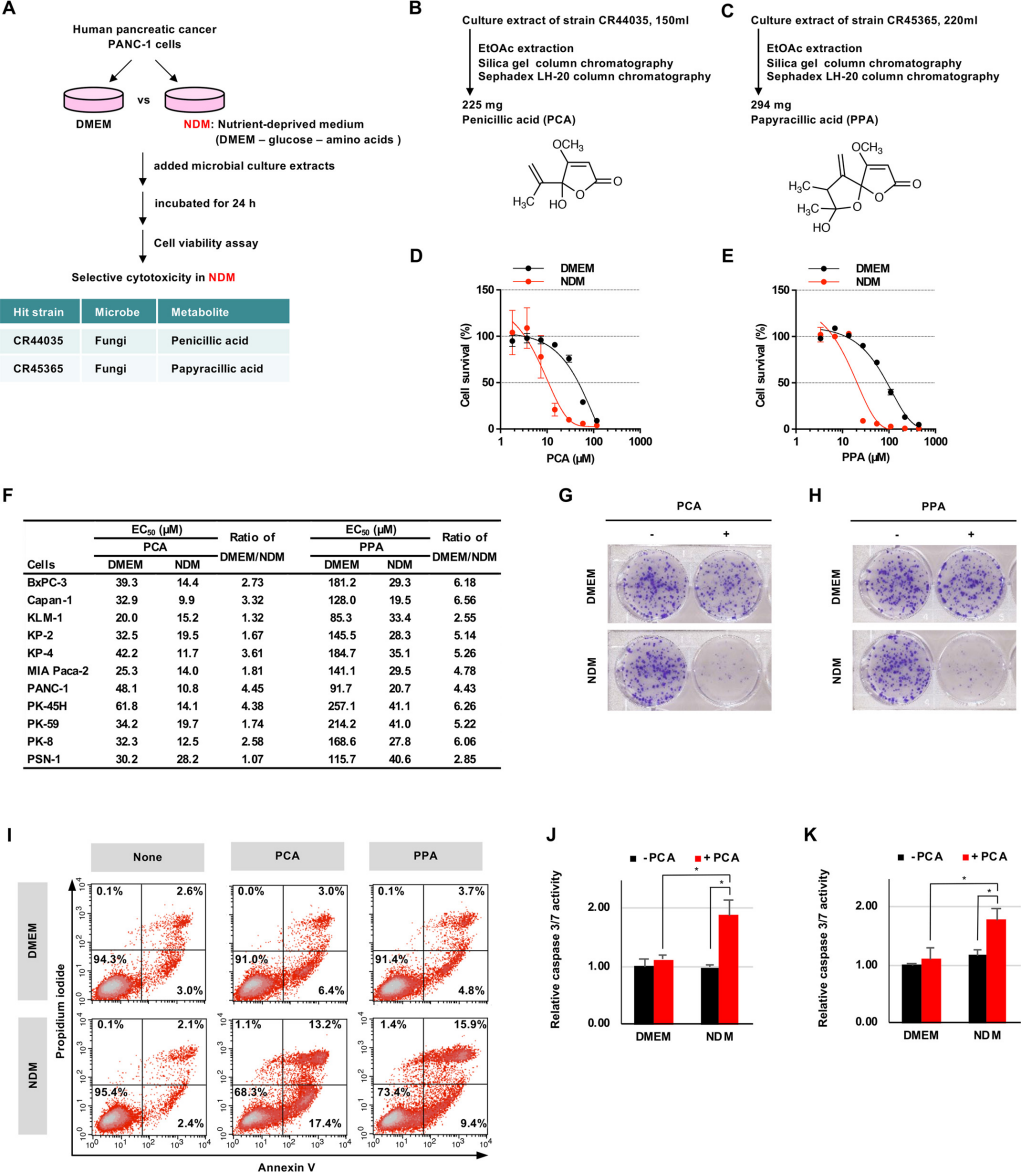
**PCA and PPA inhibit cell growth of human pancreatic cancer under nutrient-deprived conditions.***A*, schematic representation of the screening procedure for microbial metabolites that preferentially inhibit PANC-1 cell growth under nutrient-deprived conditions. *B* and *C*, isolation procedure and chemical structure of PCA (*B*) and PPA (*C*). *D* and *E*, preferential cytotoxicity of PCA (*D*) and PPA (*E*) in PANC-1 cells. PANC-1 cells were incubated with PCA (*D*) or PPA (*E*) for 24 h in NDM or DMEM. *F*, preferential cytotoxicity of PCA and PPA in human pancreatic cancer cells. *G* and *H*, colony formation of PANC-1 cells. PANC-1 cells were incubated for 10 days in DMEM after treatment with 6 μm PCA (*G*) or 4 μm PPA (*H*) for 24 h in NDM or DMEM. *I*, detection of apoptotic cells. PANC-1 cells were incubated with 30 μm PCA or 44 μm PPA for 24 h, and apoptotic cells stained with annexin V and PI were detected by flow cytometry. *J* and *K*, caspase 3/7 activity in PANC-1 cells. PANC-1 cells were incubated with 59 μm PCA (*J*) and 44 μm PPA (*K*) for 6 h. The data are presented as means ± S.D. of three independent experiments. *p* values were determined by two-tailed Student's *t* test. *, *p* < 0.05.

We constructed a high-throughput screening system to explore small molecules that preferentially reduce the survival of nutrient-deprived cancer cells (Fig. 1*A*). To identify selective cytotoxic agents that act preferentially on human pancreatic cancer PANC-1 cells grown in nutrient-deprived medium (NDM), but not in nutrient-sufficient medium (Dulbecco's modified Eagle's medium (DMEM)), we screened chemical libraries and microbial culture extracts. We found two culture extracts of fungi. The first was penicillic acid (PCA), which was isolated from a culture extract of fungal strain CR44035 (Fig. 1*B*) (28). PCA is a classical mycotoxin produced by various fungi, such as *Penicillium* and *Aspergillus* (29). The second was papyracillic acid (PPA), a PCA analog isolated from a culture extract of fungal strain CR45365 (Fig. 1*C*) (30). PCA and PPA clearly showed preferential cytotoxicity to PANC-1 cells under nutrient-deprived compared with nutrient-sufficient conditions (Fig. 1, *D* and *E*). The preferential cytotoxicity of PCA and PPA under nutrient-deprived conditions was exhibited in PANC-1 cells and other human pancreatic cancer cell lines (Fig. 1*F* and Fig. S1, A and B). We also examined cell survival under nutrient-deprived conditions in colony formation assays (Fig. 1, *G* and *H*, and Fig. S1C). PCA and PPA inhibited colony formation of PANC-1 cells under nutrient-deprived but not under nutrient-sufficient conditions. Next, we examined apoptosis induced by PCA and PPA using annexin V and propidium iodide (PI) double staining (Fig. 1*I*). PCA and PPA significantly increased the number of early (annexin V–positive/PI-negative) and late (annexin V–positive/PI-positive) apoptotic cells under nutrient-deprived conditions. We also observed PCA- and PPA-triggered activation of caspase-3/7 in only nutrient-deprived cells (Fig. 1, *J* and *K*). These results indicated that PCA and PPA preferentially inhibit cell growth of human pancreatic cancer under nutrient-deprived conditions.

### PCA and PPA bind to GSH and deplete cellular GSH

**Figure 2:**
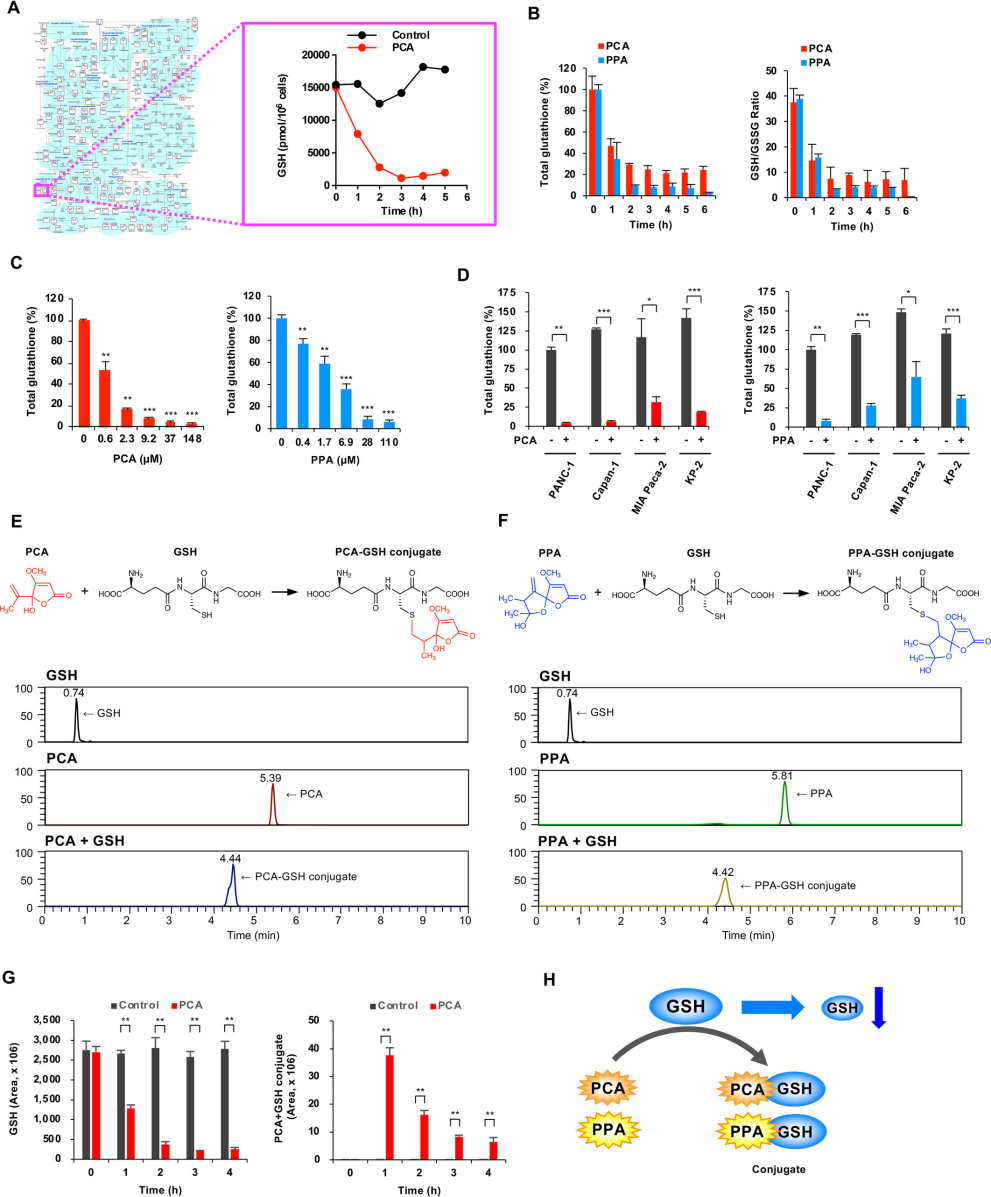
**PCA and PPA bind to GSH and decrease cellular GSH.***A*, effects of PCA on intracellular concentration of metabolites involved in the primary metabolic pathway. Intracellular metabolites of PANC-1 cells treated with 59 μm PCA were measured by CE–TOF MS. *B–D*, intracellular GSH levels. PANC-1 cells were incubated with 59 μm PCA and 88 μm PPA, and intracellular GSH levels and the GSH/GSSG ratio were measured by a GSH/GSSG-Glo assay (*B*). PANC-1 cells were incubated with various concentrations of PCA and PPA for 2 h (*C*). Human pancreatic cancer cells were treated with 59 μm PCA or 88 μm PPA for 2 h (*D*). The data are presented as means ± S.D. of three independent experiments. *p* values were determined by two-tailed Student's *t* test. *, *p* < 0.05; **, *p* < 0.01; ***, *p* < 0.001. *E*, PCA directly binds to GSH. The PCA–GSH conjugate was detected by LC–MS. *F*, PPA directly binds to GSH. *G*, formation of the PCA–GSH conjugate in PANC-1 cells. Intracellular metabolites of PANC-1 cells treated with 59 μm PCA were measured by LC–MS. The data are presented as means ± S.D. of three independent experiments. *p* values were determined by two-tailed Student's *t* test. **, *p* < 0.01. *H*, schematic model to explain the PCA- and PPA-induced decreases in GSH.

We examined the sensitivity of a panel of human cancer cell lines (JFCR39) to PCA and PPA to predict the molecular mechanism of PCA and PPA (31). Sensitivity patterns in JFCR39 were determined to be different from existing clinical drugs, indicating that PCA and PPA have unique molecular mechanisms (Fig. S2, A and B). Next, we examined metabolites of PANC-1 cells altered by PCA using capillary electrophoresis (CE)–TOF MS. In total, 218 metabolites related to primary metabolism were detected in PCA-treated PANC-1 cells. GSH, a major cellular antioxidant, was markedly decreased by PCA (Fig. 2*A*, and Fig. S2, C and D, and Table S1). Detailed analysis revealed that GSH rapidly decreases after PCA and PPA treatment and that GSH levels are less than half of its initial levels at ∼1 h (Fig. 2*B*). Similarly, the reduced GSH/GSSG ratio also decreased after PCA and PPA treatment, which depended on the PCA and PPA concentration (Fig. 2*C* and Fig. S2E). Further, we also observed such decreases in GSH after PCA and PPA treatment in Capan-1, MIA Paca-2, and KP-3 cells (Fig. 2*D* and Fig. S2F). GSH is a thiol-containing tripeptide (γ-glutamyl-cysteinyl-glycine). We monitored the direct reaction of PCA and PPA with GSH using LC–MS. We detected PCA–GSH and PPA–GSH conjugates as new peaks (Fig. 2, *E* and *F*, and Fig. S2, G and H). These data suggest that PCA and PPA form an adduct with GSH nonenzymatically. The chemical structures of PCA–GSH and PPA–GSH conjugates were assumed as shown in Fig. 2 (*E* and *F*). We used *N*-Ac-cysteine methyl ester (NACM) as a simple model compound instead of GSH in determining the detailed chemical structures of PCA–GSH or PPA–GSH conjugates (Fig. S2I). A PCA–NACM conjugate was quickly formed by incubation of PCA and NACM in PBS. However, the conjugate was a mixture of four isomers, which could not be isolated by chromatography. Using derivatization by methylation, we separated four methyl derivatives of isomers by reverse-phase HPLC and chiral HPLC (Fig. S2J). Each chemical structure was determined by analyzing NMR and MS spectroscopic data (Fig. S2, K–R).

Further, we examined PCA binding to GSH in cells. The PCA–GSH conjugate was detected in PANC-1 cells treated with PCA, indicating that PCA binds to GSH in cells as well as *in vitro* (Fig. 2*G* and Fig. S2S). These results suggested that the exomethylene groups of PCA and PPA covalently bind to the thiol group of GSH, decreasing free GSH (Fig. 2*H*).

Several small thiol metabolites exist in cells. PCA and PPA might bind to other small thiol metabolites in addition to GSH. Cys was undetectable in PANC-1 cells by LC–MS, but BxPC-3 cells contained detectable levels of Cys. Treatment of BxPC-3 cells with PCA decreased Cys levels and generated a PCA-Cys conjugate (Fig. S2, T–V). Therefore, we might also consider the relationship between PCA/PPA and other small thiol metabolites.

### PCA and PPA markedly increase ROS levels under nutrient-deprived conditions

**Figure 3:**
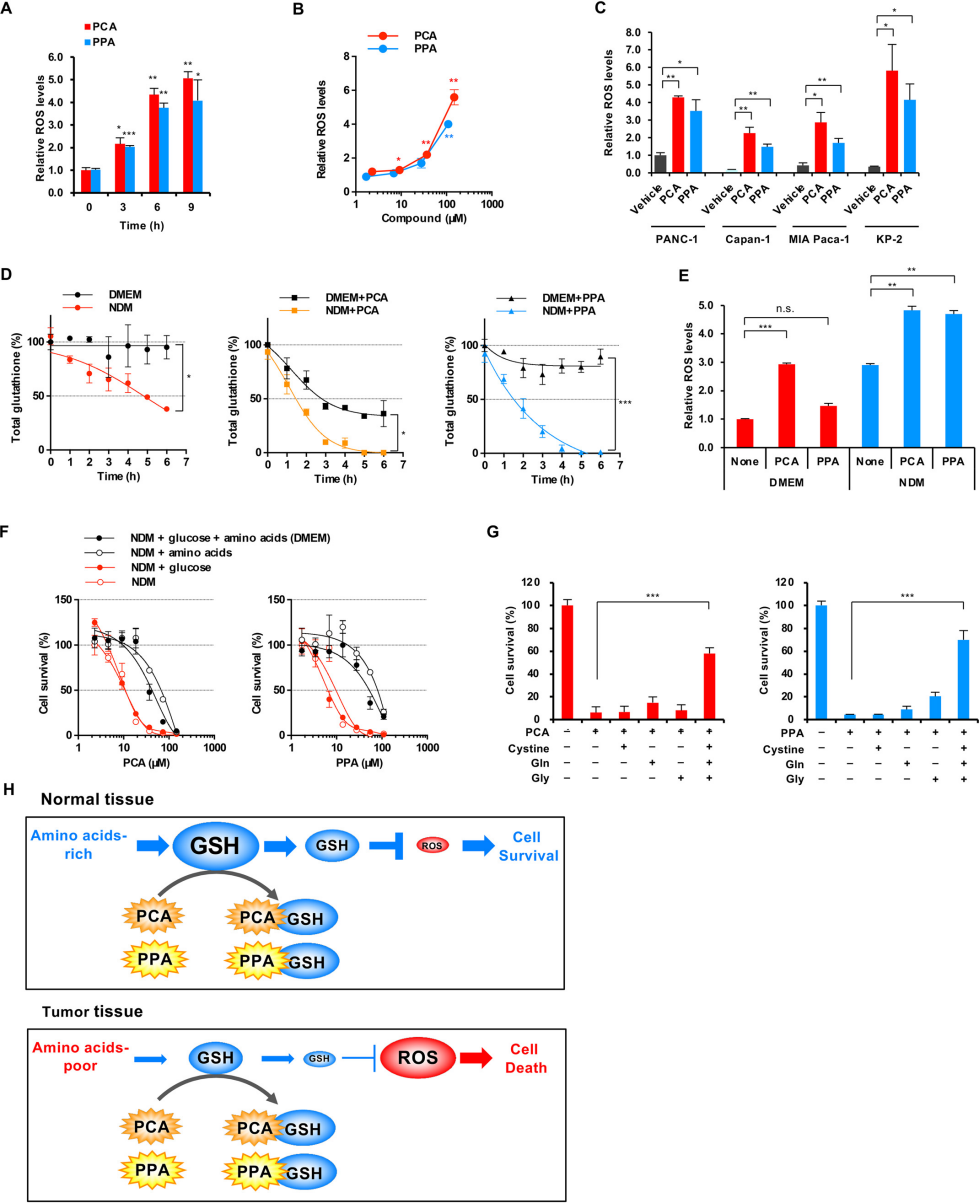
**PCA and PPA significantly increase ROS levels under nutrient-deprived conditions.***A–C*, intracellular ROS levels. PANC-1 cells were incubated with 118 μm PCA and 88 μm PPA in DMEM, and intracellular ROS levels were measured by a ROS-Glo H_2_O_2_ assay (*A*). PANC-1 cells were incubated with PCA and PPA for 12 h (*B*). PANC-1 cells were treated with 118 μm PCA or 88 μm PPA for 12 h (*C*). *D*, intracellular GSH levels under nutrient-deprived conditions. PANC-1 cells were incubated with 24 μm PCA and 18 μm PPA in NDM or DMEM. *E*, intracellular ROS levels under nutrient-deprived conditions. PANC-1 cells were incubated with 118 μm PCA and 88 μm PPA for 6 h in NDM or DMEM. *F*, effects of glucose and amino acids on preferential cytotoxicity of PCA and PPA under nutrient-deprived conditions. PANC-1 cells were incubated with PCA or PPA for 24 h in NDM to which glucose or amino acids were added. *G*, effects of cystine, glutamine, and glycine on preferential cytotoxicity of PCA and PPA under nutrient-deprived conditions. PANC-1 cells were incubated with 59 μm PCA and 44 μm PPA for 24 h in NDM with cystine, glutamine, and glycine. *H*, schematic of the mechanism underlying preferential cytotoxicity of PCA and PPA to nutrient-deprived cancer cells. For all graphs, the data are presented as means ± S.D. of three independent experiments. *p* values were determined by two-tailed Student's *t* test. *, *p* < 0.05; **, *p* < 0.01; ***, *p* < 0.001; *n.s.*, not significant.

PCA and PPA decrease intracellular GSH levels, shifting intracellular ROS generation–antioxidant balance toward an increase in ROS levels. PCA and PPA treatment dose- and time-dependently increased intracellular ROS levels in PANC-1 cells, as shown by intracellular H_2_O_2_ concentrations (Fig. 3, *A* and *B*). This increase was observed not only in PANC-1 cells but also in Capan-1, MIA Paca-2, and KP-3 cells (Fig. 3*C*). These results indicated that PCA and PPA decrease GSH and increase ROS levels, leading to apoptosis.

Investigation of the relationship between nutrient deprivation and GSH levels showed that intracellular GSH in PANC-1 cells gradually decreases under nutrient-deprived conditions, but nutrient-sufficient conditions, understandably, did not affect GSH levels (Fig. 3*D*). PCA and PPA decreased GSH levels more rapidly under nutrient-deprived compared with nutrient-sufficient conditions. Investigation of the relationship between nutrient deprivation and ROS levels showed that intracellular ROS levels are higher in nutrient-deprived cells than in nutrient-sufficient cells at basal levels (Fig. 3*E*), indicating that nutrient deprivation increases oxidative stress in cancer cells. In addition, PCA and PPA treatment induced large accumulation of ROS in nutrient-deprived compared with nutrient-sufficient cells, which might contribute to increased apoptosis when nutrients are deemed insufficient. However, preferential cytotoxicity under nutrient-deprived conditions by PCA and PPA remains to be elaborated.

We investigated the effects of nutritional composition on PCA and PPA cytotoxicity under nutrient-deprived conditions. Preferential cytotoxicity of PCA and PPA was induced by deprivation of amino acids, not glucose (Fig. 3*F*). NDM lacks 15 amino acids available in DMEM. We investigated the effect of 15 amino acids on PCA cytotoxicity in NDM. Single amino acid supplementation could not rescue PCA cytotoxicity (Fig. S3A). However, simultaneous addition of Gln, Gly, and Cys (precursor amino acids of GSH) abrogated PCA preferential cytotoxicity under nutrient-deprived conditions (Fig. 3*G*). The decrease in cellular GSH by nutrient deprivation can be attributed to a limitation of the supply of precursor amino acids of GSH.

Consequently, we propose the molecular mechanism of PCA and PPA (Fig. 3*H*). Tumor tissue shows reduced content of glucose and amino acids because of the aberrant cell proliferation and abnormal blood vessels, which decrease GSH levels. PCA and PPA nonenzymatically bind to depleted GSH, further decreasing GSH levels. The resulting small amount of GSH can no longer prevent cellular damage caused by ROS, often resulting in induction of apoptosis. In contrast, nutrient-sufficient cells can produce sufficient GSH in normal tissue. Even if PCA and PPA decrease GSH, the remaining GSH will be enough to control redox status and prevent cellular damage by ROS.

### Auranofin preferentially inhibits the growth of human pancreatic cancer under nutrient-deprived conditions

**Figure 4:**
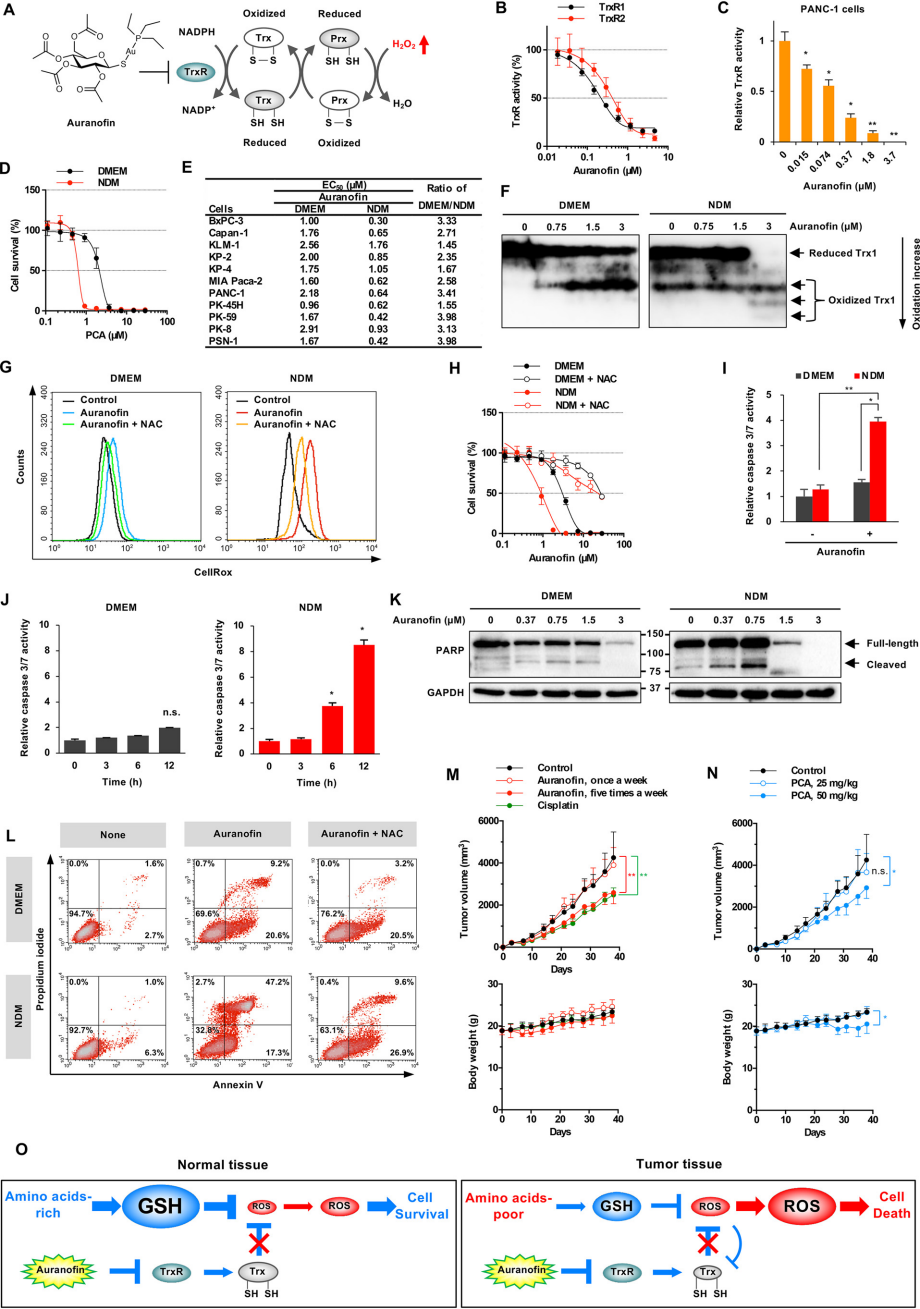
**Auranofin preferentially inhibits cell growth of human pancreatic cancer under nutrient-deprived conditions.***A*, chemical structure of auranofin and TrxR inhibition. *B*, auranofin inhibits human recombinant TrxR1/2 activity. *C*, auranofin inhibits TrxR activity in PANC-1 cells. The data are presented as means ± S.D. of three independent experiments. *p* values were determined by two-tailed Student's *t* test. *, *p* < 0.05; **, *p* < 0.01. *D*, preferential cytotoxicity of auranofin in PANC-1 cells. PANC-1 cells were incubated with auranofin for 24 h in NDM or DMEM. *E*, preferential cytotoxicity of auranofin in human pancreatic cancer PANC-1 cells. *F*, redox state of Trx1 in PANC-1 cells under nutrient-deprived conditions. The redox state was examined by redox Western blotting. *G*, intracellular ROS levels. PANC-1 cells were treated with 1.5 μm auranofin for 12 h in the absence or presence of 5 mm NAC. *H*, NAC abrogated the inhibition of cell growth by auranofin. PANC-1 cells were incubated with auranofin for 24 h in the absence or presence of 5 mm NAC. *I* and *J*, caspase 3/7 activity in PANC-1 cells. PANC-1 cells were incubated with 1.5 μm auranofin for 12 h (*I*) or the indicated time (*J*). The data are presented as means ± S.D. of three independent experiments. *p* values were determined by two-tailed Student's *t* test. *, *p* < 0.05; **, *p* < 0.01; ***, *p* < 0.001; *n.s*., not significant. *K*, PARP activation. PANC-1 cells were treated with auranofin for 24 h. *L*, detection of apoptotic cells. PANC-1 cells were incubated with 0.75 μm auranofin for 12 h in the absence or presence of 5 mm NAC. *M*, antitumor activity of auranofin. Auranofin (12.5 mg/kg, intraperitoneally, *n* = 5) or cisplatin (12.5 mg/kg, intravenously, once a week, *n* = 5) was administered to mouse xenograft models of human PSN-1 cancer. *p* values were determined by two-tailed Student's *t* test. **, *p* < 0.01. *N*, antitumor activity of PCA. PCA (12.5 or 50 mg/mkg, *n* = 5) was administered intraperitoneally once a week to PSN-1 tumor-bearing mice. *p* values were determined by two-tailed Student's *t* test. *, *p* < 0.05; *n.s.*, not significant. *O*, schematic of the mechanism underlying preferential cytotoxicity of auranofin to nutrient-deprived cancer cells.

Small molecular compounds that decrease antioxidant GSH, such as PCA and PPA, show selective cytotoxicity to human pancreatic cancer cells under nutrient-deprived conditions. Trx serves a partially overlapping and complementary role to GSH for protection from oxidative stress (32). Small molecular compounds that decrease Trx levels might also show such selective cytotoxicity. Auranofin is an Au(I) complex containing an gold–sulfur bond stabilized by a triethylphosphine group. This agent inhibits cytosolic Trx reductase 1 (TrxR1) and mitochondrial TrxR2, thus decreasing the reduced form of Trx (Fig. 4*A*) (33, 34). Auranofin markedly inhibited TrxR activities of human recombinant TrxR1 and TrxR2 proteins (Fig. 4*B*). In addition, auranofin inhibited total intracellular TrxR activity in PANC-1 and PSN-1 cells (Fig. 4*C* and Fig. S4A), and auranofin treatment showed preferential cytotoxicity under nutrient-deprived compared with nutrient-sufficient conditions in PANC-1 cells and other human pancreatic cancer cells (Fig. 4, *D* and *E*, and Fig. S4B). Furthermore, preferential cytotoxicity of auranofin was induced by deprivation of amino acids, but not glucose (Fig. S4C). Auranofin might cause an imbalance between reduced and oxidized Trx, because alteration of the intracellular redox state triggers tumor cell apoptosis (35).

We have examined the redox states of Trx in PANC-1 cells incubated with auranofin using modified redox Western blotting (Fig. 4*F*) (36, 37). The uppermost band indicates the fully reduced form of Trx, whereas the more oxidized forms appeared in the lower part of the gel. At basal levels, we observed the oxidized forms of Trx in nutrient-deprived compared with nutrient-sufficient cells. Auranofin treatment obviously dose-dependently increased the oxidized forms of Trx under nutrient-deprived conditions. In parallel, auranofin treatment increased ROS levels under nutrient-deprived conditions in PANC-1, MIA Paca-2, and PSN-1 cells (Fig. 4*G* and Fig. S4D). However, the same treatment did not increase ROS levels in KP-4 cells that display only a small difference in cytotoxicity in DMEM *versus* NDM (Fig. 4*E*). Moreover, auranofin-induced ROS generation was eliminated by treatment with an antioxidant, such as *N*-acetyl-l-cysteine (NAC). NAC treatment also counteracted auranofin cytotoxicity (Fig. 4*H* and Fig. S4E). Therefore, accumulation of ROS can be attributed to auranofin-induced cytotoxicity.

We also examined caspase 3/7 activity, activation of poly(ADP-ribose) polymerase (PARP), and occurrence of apoptotic cells to assess auranofin impact on apoptosis. Auranofin significantly up-regulated caspase 3/7 activity in PANC-1 cells time-dependently under nutrient deprivation (Fig. 4, *I* and *J*). Caspase 3/7 activation subsequently induced proteolytic cleavage of PARP, and finally, underwent apoptosis. Auranofin treatment in nutrient-deprived cells clearly showed activation of PARP cleavage (Fig. 4*K*). The percentage of apoptotic cells (annexin V and PI double-positive) were significantly increased by auranofin treatment under nutrient-deprived conditions (Fig. 4*L*). In addition, NAC was able to reduce the percentage of auranofin-induced apoptotic cells.

Finally, we evaluated the antitumor activity of auranofin using a nude mouse xenograft model of human pancreatic cancer. Intraperitoneal administration of auranofin showed significant suppression of tumor growth in PSN-1 cancers. Auranofin suppressed tumor growth up to ∼40% of vehicle control, and its antitumor activity was comparable with that of cisplatin (Fig. 4*M*). We found no difference in body weight between auranofin-treated and control mice after treatment, which had no severe side effects. We also evaluated the anti-tumor activity of PCA on PSN-1 tumor-bearing mice. PCA suppressed tumor growth up to ∼30% of vehicle control (Fig. 4*N*). Next, we investigated the combined effect of PCA/auranofin and cisplatin. Combination therapy has been considered as an effective tool for augmenting efficacy, preventing the development of drug resistance, and reducing treatment duration. PCA/PPA is expected to increase the sensitivity of pancreatic cancer cells to cisplatin because cisplatin is inactivated by GSH (38, 39). PCA/PPA/auranofin showed increased efficacy in colony formation assays and 3D spheroid models (Fig. S4, F–H). Unfortunately, PCA and auranofin did not show this effect with cisplatin on *in vivo* mouse xenografts (Fig. S4, I and J). A more detail investigation will be required to enhance a combined effect with cisplatin.

Accordingly, we propose a molecular mechanism of auranofin action under nutrient-deprived conditions (Fig. 4*O*). Tumor tissue tends to show reduced GSH levels because of a deficient supply of amino acids. Auranofin inhibits TrxR and suppresses production of the reduced form of Trx regardless of nutrient conditions. Auranofin induces accumulation of ROS under nutrient deprivation, eventually leading to cell death. In contrast, under nutrient-sufficient conditions, such as in normal tissues, the accumulation of ROS by auranofin is not enough to induce apoptosis. Therefore, nutrient-deprived cancer cells show greater vulnerability to auranofin.

## Discussion

Tumor microenvironments, such as local nutrient deprivation, are involved in tumor progression. Cytotoxic agents that act preferentially under nutrient-deprived conditions could function as promising antitumor agents. PCA and PPA show preferential cytotoxicity under nutrient-deprived conditions. PCA was identified in 1936 as a mycotoxin produced by *Penicillium puberulum* and *Penicillium cylopium* (28). PCA possesses a variety of biological properties, such as antitumor activity, antiviral activity, inhibition of NF-κB activation, and inhibition of Fas ligand–induced apoptosis (40, 41, 42). In addition, PCA forms a conjugate with Cys and GSH nonenzymatically (43). PPA, a PCA analog produced by the ascomycete *Lachnum papyraceum*, is known to possess antibacterial, antifungal, and phytotoxic activities (30, 44). PPA also reacts with Cys, forming a PPA–Cys conjugate via covalent binding between the exomethylene group of PPA and the thiol group of Cys (45). Exomethylene groups of PCA and PPA are predicted to bind covalently to the thiol group of GSH, but the detailed chemical structures of conjugates have not been determined yet. GSH conjugates are a mixture of isomers and have complex NMR spectra. We used NACM as a simple model compound instead of GSH, isolated isomers of PCA–NACM conjugates, and determined chemical structures.

GSH is considered a primary target of PCA and PPA, and GSH levels are closely related to the elevated sensitivity of nutrient-deprived cancer cells to PCA and PPA. This idea is supported by our observation that complementation of precursor amino acids of GSH abrogates the sensitivity to PCA and PPA. In addition, our results are consistent with previous studies showing that cotreatment with sulfasalazine (an inhibitor of the cystine–glutamate antiporter) and buthionine-(*S*,*R*)-sulfoximine (an inhibitor of a rate-limiting enzyme for GSH synthesis) synergistically induce cancer cell death (46). Therefore, targeting redox systems, such as GSH, might be an effective therapeutic strategy in some tumor microenvironments. However, PCA and PPA show differential effects on several cell lines. We might also consider the possibility that PCA and PPA affect the function of Cys-containing proteins and other small thiol metabolites in addition to GSH, because PCA and PPA react to these proteins and metabolites.

Auranofin, originally developed as a therapeutic agent for rheumatoid arthritis (47), inhibits TrxR, the main functional component of the Trx system (33, 34). TrxR plays a key role in converting the oxidized form of Trx (Trx-S_2_) to its reduced form (Trx-(SH)_2_). Auranofin shows preferential cytotoxicity to nutrient-deprived cancer cells, similar to PCA and PPA. Further, auranofin induces apoptosis by increasing ROS levels and altering the cellular redox status. Moreover, auranofin suppresses pancreatic tumor growth in a xenograft model. Auranofin has recently been investigated as an antitumor agent (48) and showed anti-tumor activities in non–small-cell lung cancer and osteosarcoma xenografts (49, 50). These results are in agreement with our observations. Therefore, both GSH and Trx redox systems could be therapeutic targets for the treatment of malignant tumors.

Many cancer cells are often exposed to chronic hypoxia and nutrient deprivation, which are caused by inadequate blood supply through tumor blood vessels. In particular, pancreatic cancer, characterized by poor vascularization and high interstitial pressure, shows high levels of hypoxia (8, 9). Metabolomic comparisons of human pancreatic tumors and adjacent normal tissues have revealed that tumor tissue is low in glucose and certain amino acids (7, 51). Depletion of amino acids impairs GSH synthesis and decreases GSH levels in tumor tissues. GSH levels are not yet clearly defined in pancreatic cancer, but brain and liver tumors show low GSH levels (52). A decrease in GSH levels using chemical inhibitors or genetic loss of GSH synthesis enhances sensitivity to TrxR inhibition (46). Therefore, redox system inhibitors, such as PCA, PPA, and auranofin, could be highly effective against tumors with low GSH levels.

Current bioinformatic analysis has revealed that *ARID1A* encodes an SWI/SNF chromatin-remodeling factor that is frequently mutated in a variety of cancers (53, 54). Especially, 57% of ovarian clear-cell carcinomas, an aggressive human cancer, had *ARID1A* mutations (55). *ARID1A* promotes the expression of SLC7A11, which encodes a subunit of the cystine/glutamate antiporter xCT. SLC7A11 expression is lower in *ARID1A*-deficient compared with *ARID1A*-proficient cancer cells, contributing to a decrease in basal GSH levels and an increase in ROS in *ARID1A*-deficient cells (56). Ogiwara *et al.* (56) showed that *ARID1A*-deficient cancer cells are sensitive to small molecule APR-246, which is converted to the Michael acceptor methylene quinuclidinone that reacts directly with the thiol group of GSH. Although APR-246 covalently binds to cysteine residues in multiple polypeptides, GSH is considered a major target of APR-246. The mode of action of APR-246 is closely similar to that of PCA and PPA. These latter molecules might also be highly effective toward *ARID1A*-deficient cancer cells.

In this study, we demonstrated that nutrient-deficient cancer cells are susceptible to inhibitors of redox systems, such as PCA, PPA, and auranofin, using mainly human pancreatic cancer PANC-1 cells; use of other cell lines is specifically mentioned in the text. Redox systems are identified to play an essential role in maintaining homeostasis and promoting cell survival of pancreatic cancers, making them attractive therapeutic targets for malignant tumors. Inhibitors of redox systems might be useful clinically as unique therapeutic agents against human pancreatic cancers.

## Experimental procedures

### Cell culture

PANC-1, Capan-1, MIA Paca-2, and BxPC-3 cells were purchased from the ATCC; KP-2 and KP-3 cells from the Japanese Collection of Research Bioresources Cell Bank; PSN-1 cells from the European Collection of Authenticated Cell Cultures; and KLM-1, PK-45H, PK-59, KP-4, and PK-8 cells from the RIKEN Bioresource Research Center through the National Bio-Resource Project of the Ministry of Education, Culture, Sports, Science and Technology of Japan. These cells were routinely cultured in DMEM (Nissui, Tokyo, Japan) supplemented with 10% fetal bovine serum (Cosmo Bio, Tokyo, Japan), 100,000 units/liter of penicillin G, and 100 mg/liter of streptomycin at 37 °C in a humidified incubator containing a 5% CO_2_ atmosphere. The cells were authenticated using short tandem repeat analysis (GlobalFilter PCR amplification kit; Thermo Fisher Scientific, San Jose, CA). *Mycoplasma* testing was performed using a PCR mycoplasma detection set (Takara Bio Inc., Shiga, Japan).

Nutrient deprivation was achieved by culturing cells in NDM, as previously described (57). Briefly, NDM comprised 265 mg/liter of CaCl_2_·H_2_O, 400 mg/liter of KCl, 200 mg/liter of MgSO_4_·7H_2_O, 6400 mg/liter of NaCl, 163 mg/liter of NaH_2_PO_4_·2H_2_O, 0.1 mg/liter of Fe(NO_3_)_3_·9H_2_O, 5 mg/liter of phenol red, 100,000 units/liter of penicillin G, 100 mg/liter of streptomycin, 25 mmol/liter of HEPES buffer (pH 7.4), a MEM vitamin solution (Thermo Fisher Scientific), and 10% dialyzed fetal bovine serum. The final pH was adjusted to 7.4 with 10% NaHCO_3_. NDM was then supplemented with 1 g/liter of d-glucose and/or amino acids (42 mg/liter of l-serine, 30 mg/liter of glycine, 292 mg/liter of l-glutamine, and 2% MEM amino acid solution; Thermo Fisher Scientific) for some experiments.

### Preferential cytotoxicity under nutrient-deprived conditions

PANC-1 cells were seeded into 96-well plates at a density of 2.5 × 10^4^ cells/well and incubated overnight in DMEM. Then the cells were washed with PBS, and the medium was replaced with either fresh DMEM or NDM. Microbial culture extracts or chemicals were added into each well, and the cells were incubated for an additional 24 h. Next, the medium was replaced with DMEM containing 0.5 mg/ml of thiazolyl blue tetrazolium bromide (Dojindo Laboratory, Inc., Kumamoto, Japan) and incubated for 4 h. The cells were supplemented with 20% SDS and incubated at 37 °C for 12 h. Absorbance was measured at a wavelength of 570 nm using a microplate reader.

### Fermentation and isolation of PCA and PPA

PCA was produced by the fungal strain CR44035. A slant culture of CR44035 was inoculated into a 100-ml Erlenmeyer flask containing 20 ml of seed medium comprising 2.0% soluble starch, 1.0% glucose, 0.5% polypeptone, 0.6% wheat germ, 0.3% yeast extract, and 0.2% CaCO_3_ in deionized water; the pH was adjusted to 7.0 with NaOH solution before sterilization. The flask was incubated at 25 °C for 4 days on a rotary shaker at 220 rpm. Aliquots (3 ml) of this seed culture were transferred into 500-ml Erlenmeyer flasks containing a mixture of 2 g of oatmeal and 80 g of water-absorbed rice as a solid production medium. The flasks were cultured at 25 °C for 14 days without shaking. Then 400 g of the obtained culture was extracted with 800 ml of 67% aqueous acetone and was later filtered. The filtrate was concentrated to 150 ml under reduced pressure, and the resulting residue was partitioned between 100 ml of H_2_O and 250 ml of ethyl acetate. The organic fraction was dried over Na_2_SO_4_, filtered, and concentrated under reduced pressure. The resulting residue was chromatographed using a silica gel column with CHCl_3_–MeOH to obtain a crude material. Finally, this material was applied to a Sephadex LH-20 column (GE Healthcare) to obtain 225 mg of pure PCA.

PPA was produced by the fungus strain CR45365. Solid culture was carried out by the same procedure as PCA fermentation. The obtained culture extract (220 ml) was partitioned with 220 ml of ethyl acetate. The organic fraction was dried over Na_2_SO_4_, filtered, and concentrated under reduced pressure, and the resulting residue was chromatographed using a silica gel column with CHCl_3_–MeOH to obtain a crude material. Finally, this material was applied to a Sephadex LH-20 column (GE Healthcare) to obtain 294 mg of pure PPA.

### Colony formation assay

PANC-1 cells were seeded in 6-well plates at a density of 4 × 10^2^ cells/well and incubated for 24 h. The cells were washed with PBS, and the medium was replaced with either fresh DMEM or NDM. Next, 6 μm PCA or 4 μm PPA was added into each well, and the cells were incubated for an additional 24 h. Then the medium was replaced with DMEM after washing with PBS, and the cells were incubated for 10 days. Finally, the colonies were washed twice with PBS, fixed with 2.5% glutaraldehyde in PBS, and stained with 0.4% crystal violet solution in 20% MeOH.

### Annexin V/propidium iodide assay

Apoptosis of PANC-1 cells was measured using an annexin V–FITC apoptosis detection kit (BioVision, Inc., Milpitas, CA) according to the manufacturer's instructions. Briefly, PANC-1 cells were incubated with annexin V–FITC and PI and then analyzed using a FACSCalibur flow cytometer (BD Biosciences, Franklin Lakes, NJ). The percentages of early (annexin V–positive, PI-negative) and late (annexin V–positive, PI-positive) apoptotic cells were quantified using BD Cell Quest Pro™ version 5.2.1 (BD Biosciences).

### Caspase 3/7 activity

Caspase 3/7 activity was measured using the caspase 3/7-Glo Assay (Promega, Madison, WI) according to the manufacturer's instructions. Luminescence was measured using an EnSpire multimode plate reader (PerkinElmer).

### Metabolome analysis

PANC-1 cells (2 × 10^6^) were preincubated in 6 mm dishes in DMEM for 24 h. The culture medium was replaced with fresh DMEM, 59 μm PCA was added to each dish, and the cells were incubated for up to 5 h. The culture medium was removed from each dish, and the cells were washed twice with 5 ml and then 1 ml of 5% mannitol solution. Next, the cells were treated with 400 μl of methanol and 275 μl of Milli-Q water containing internal standards (H3304-1002, Human Metabolome Technologies, Tsuruoka, Yamagata, Japan). The extract was transferred to a microcentrifuge tube and centrifuged at 2300 × *g* at 4 °C for 5 min, and 500 μl of the upper aqueous layer was centrifugally filtered at 9100 × *g* at 4 °C for 120 min through a Millipore 5-kDa cutoff filter (Ultrafree MC PLHCC, Human Metabolome Technologies). The obtained filtrates containing metabolites were measured using CE–TOF MS. Metabolome analysis was conducted using the Basic Scan package of Human Metabolome Technologies with CE–TOF MS, as described previously (58, 59).

GSH and PCA–GSH conjugates were also assayed using LC–MS (Q Exactive hybrid quadrupole-Orbitrap mass spectrometer with an UltiMate 3000 HPLC system, Thermo Fisher Scientific). LC–MS analysis was performed using the following conditions: a 3-μm, 2.1 × 150-mm Discovery HS F5-3 (Sigma–Aldrich); a flow rate of 0.25 ml/min; a solvent system comprising solvent A (water containing 0.1% formic acid) and solvent B (acetonitrile containing 0.1% formic acid); a gradient program comprising 100% solvent A at 0 min, 100% solvent A at 2 min, 25% solvent A and 75% solvent B at 5 min, 35% solvent A and 65% solvent B at 11 min, 95% solvent A and 5% solvent B at 15 min, and 95% solvent A and 5% solvent B at 20 min; a mass spectrometer, a positive ion; and detection of protonated molecules, *m/z* 308.0911 ± 0.0009 for GSH, and *m/z* 478.1490 ± 0.0014 for the PCA–GSH conjugate.

### GSH and GSSG levels

GSH levels and the GSH/GSSG ratio were measured using a GSH/GSSG-Glo Assay (Promega) according to the manufacturer's instructions. Luminescence was measured using an EnSpire multimode plate reader (PerkinElmer).

### Reaction of PCA and PPA with GSH

The direct reaction of PCA and PPA with GSH was detected using LC–MS. Briefly, 1 mm PCA or 1 mm PPA was incubated with 1 mm GSH in 100 μl of PBS at room temperature for 24 h. The reaction was monitored by LC–MS. LC–MS analysis was performed under the following conditions: a 3-μm, 2.0 × 50-mm Capcell Pak C18 MG III column (Osaka Soda Co. Ltd., Osaka, Japan); a flow rate of 0.2 ml/min; a solvent system comprising solvent A (water containing 0.1% formic acid) and solvent B (acetonitrile containing 0.1% formic acid); a gradient program comprising 95% solvent A and 5% solvent B at 0 min, 95% solvent A and 5% solvent B at 1 min, 100% solvent B at 10 min, and 100% solvent B at 15 min; a mass spectrometer, a positive ion; and detection of protonated molecules, *m/z* 308.0911 ± 0.0009 for GSH, *m/z* 171.0652 ± 0.0005 for PCA, *m/z* 227.0914 ± 0.0007 for PPA, *m/z* 478.1490 ± 0.0014 for the PCA–GSH conjugate, and *m/z* 534.1752 ± 0.0016 for the PPA–GSH conjugate.

### Structure elucidation of the PCA–NACM conjugate

PCA–NACM conjugates were isolated by derivatization and HPLC (Fig. S2G). Briefly, 100 mg of 0.59 mmol PCA was incubated with 190 mg of 1.12 mmol NACM in 6 ml of PBS at room temperature for 3 h. The reaction mixture was concentrated under reduced pressure, and the resulting residue was purified using reversed-phase HPLC with a 5 μm, 30 × 250 mm^2^ Capcell Pak UG120 column (Osaka Soda) and a solvent (20% acetonitrile containing 0.1% acetic acid) at a flow rate of 20 ml/min to obtain 201.3 mg of a 0.58 mmol PCA–NACM conjugate mixture. Then 2 ml of trimethylsilyldiazomethane (0.6 m in hexane) was added to a solution of 105.8 mg of PCA–NACM conjugates in 4 ml of methanol, and the mixture was stirred at room temperature for 1 h. Next, the reaction mixture was concentrated under reduced pressure, and the resulting residue was purified using reversed-phase HPLC with a 5-μm, 30 × 250-mm^2^ Capcell Pak UG120 column (Osaka Soda) and a solvent (25% acetonitrile containing 0.1% acetic acid) at a flow rate of 20 ml/min to obtain three fractions. Fraction 1 contained 4.7 mg of isomer **1** of a methyl derivative of the PCA–NACM conjugate. The chemical structure of isomer **1** was determined by analysis of NMR and MS spectroscopic data (Fig. S2, H and I). Fraction 2 contained 6.0 mg of isomer **2** (Fig. S2, J and K). In addition, a mixture of isomers **3** and **4** was isolated by chiral HPLC with a 4.6 × 250 mm^2^ Chiralpak IG column (DAICEL Corp., Tokyo, Japan) and a solvent (80% hexane containing 20% 2-propanol) at a flow rate of 1 ml/min to obtain 5.4 mg of pure isomer **3** (Fig. S2, L and M) and 4.2 mg of isomer **4** (Fig. S2, N and O). Each isomer was successfully isolated, but their absolute configurations have not yet been determined.

### ROS levels

Intracellular ROS levels were measured using a ROS-Glo H_2_O_2_ assay (Promega) and CellROX Green reagent as a fluorogenic probe (Invitrogen). Luminescence was measured using an EnSpire multimode plate reader (PerkinElmer). CellROX Green–labeled PANC-1 cells were analyzed using a FACSCalibur flow cytometer (BD Biosciences).

### TrxR activity

TrxR activity was assessed using a thioredoxin reductase assay kit (Cayman Chemical, Ann Arbor, MI, USA) according to the manufacturer's instructions. Auranofin was purchased from FUJIFILM Wako Pure Chemical Corporation (Osaka, Japan).

### Redox Western blotting

The intracellular Trx1 redox state were determined using redox Western blotting (37, 60). Briefly, the redox state of proteins obtained from PANC-1 cells treated with auranofin for 24 h was determined, as described previously by Folda *et al.* (36) with modifications. Aliquots of protein extracts were separated using urea–PAGE (6 m urea and 7.5% acrylamide) under nonreducing conditions, blotted to a nitrocellulose membrane, and probed for Trx1 using an anti-Trx1 primary antibody (catalog no. 2429; Cell Signaling Technology, Danvers, MA, USA) and anti–rabbit horseradish peroxidase–linked sheep secondary antibody (GE Healthcare). The blots were detected by enhanced chemiluminescence (ECL; GE Healthcare) and quantified using a LAS-1000 luminescent image analyzer (FUJIFILM Corp., Tokyo, Japan).

### Western blotting

Equal protein amounts were separated by SDS-PAGE and transferred to polyvinylidene difluoride membranes (Millipore, Bedford, MA, USA). The membranes were incubated with primary antibodies against PARP (catalog no. 9532; Cell Signaling Technology) and glyceraldehyde 3-phosphate dehydrogenase (sc-47724; Santa Cruz Biotechnology, Dallas, TX) for 1 h at room temperature. Primary antibodies were detected using either an anti-mouse or anti-rabbit horseradish peroxidase–linked sheep secondary antibody (GE Healthcare). The blots were later developed with ECL reagent according to the manufacturer's instructions (GE Healthcare).

### Animal experiments

All mouse experiments were conducted in accordance with the code of practice established by the ethical committee of the Microbial Chemistry Research Foundation. 5-week-old female BALB/c nude mice were purchased from Charles River Japan (Yokohama, Japan). The mice were maintained in a specific pathogen-free barrier facility in accordance with our institutional guidelines. Then 1 × 10^7^ PSN1 cells were injected subcutaneously into the left lateral flank of each mouse. 3 days after inoculation, the mice were randomly divided into three groups: control (*n* = 9), auranofin (*n* = 5), and cisplatin (*n* = 5). Next, 12.5 mg/kg of auranofin was administered to the mice intraperitoneally 5 times weekly for 5 weeks, and 2.5 mg/kg of cisplatin was administered intravenously once weekly for 5 weeks. Tumor dimensions were measured using calipers, and the tumor volume was estimated using the following formula: tumor volume = (length × width^2^)/2.

### Statistical analysis

The data are expressed as the means ± S.D. Statistical analyses were performed using Microsoft Excel and GraphPad Prism 6 (GraphPad Software Inc., San Diego, CA, USA). Statistical significance was evaluated using the two-tailed Student's *t* test, and statistically significant differences are indicated by *asterisks* as follows: *, *p* < 0.05; **, *p* < 0.01; ***, *p* < 0.001; *n.s.*, not significant.

## Data availability

All data are contained within the article.
