# Supplementary material for: Human pancreatic cancer cells under nutrient deprivation are vulnerable to redox system inhibition
Source: J Biol Chem. 2021 Jan 13;295(49):16678–90. doi: 10.1074/jbc.RA120.013893 (PMC7864064; doi:10.1074/jbc.RA120.013893)
Supplement: Supplementary file 1 [file mmc1.zip › 160180_2_supp_598561_qgscq1.pdf]

Supporting Information

Human pancreatic cancer cells under nutrient deprivation are vulnerable to redox system inhibition

Takefumi Onodera[1†], Isao Momose[1†]*, Hayamitsu Adachi[1], Yohko Yamazaki[1], Ryuichi Sawa[2], Shun-ichi Ohba[1], and Manabu Kawada[1]

[1]Institute of Microbial Chemistry (BIKAKEN), Numazu, Shizuoka 410-0301, Japan

[2]Institute of Microbial Chemistry (BIKAKEN), Tokyo 141-0021, Japan

[†]Both authors contributed equally to this work.
*Corresponding author: Isao Momose
E-mail: imomose@bikaken.or.jp

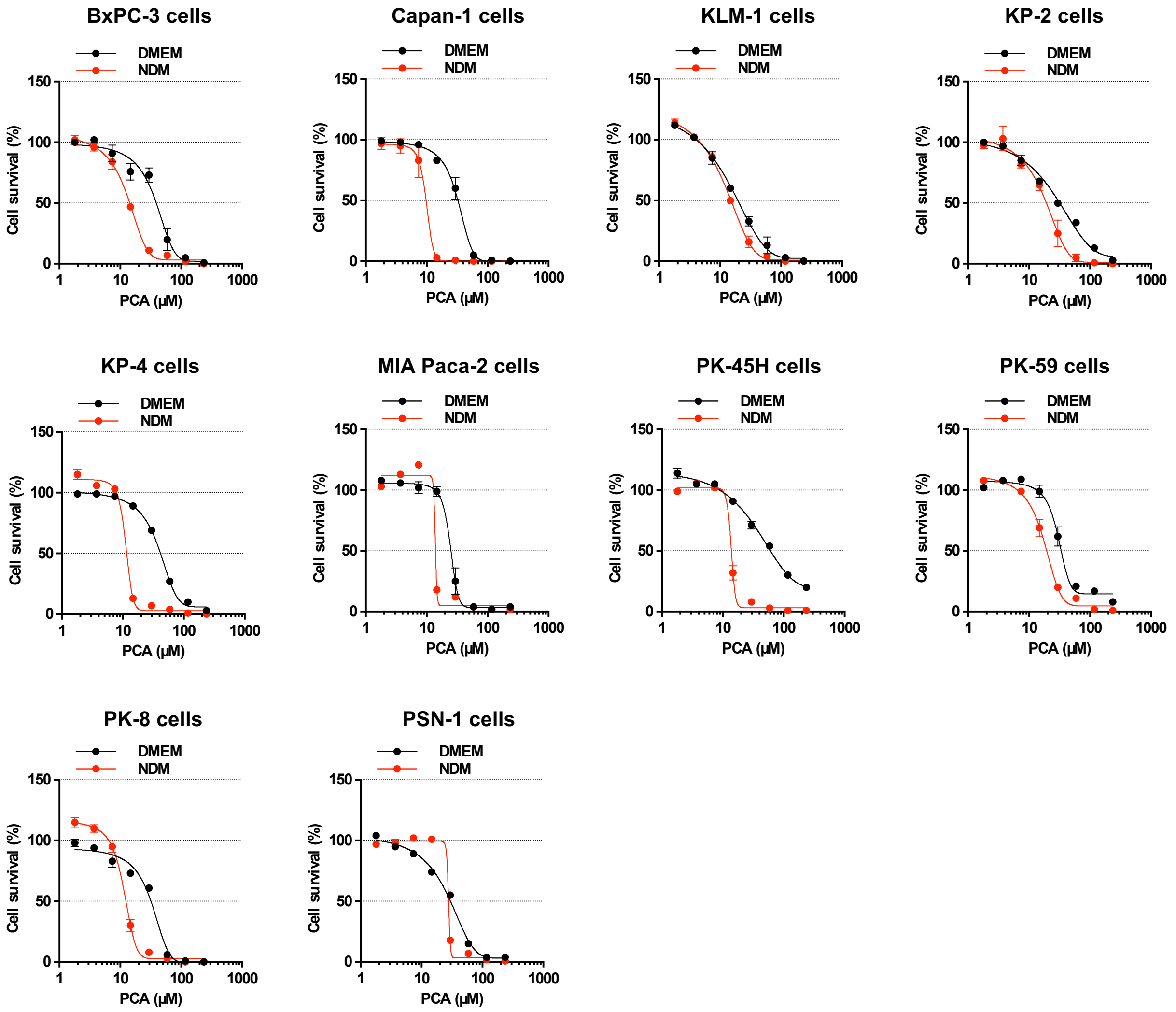

SUPPLEMENTAL FIGURE S1A. **Preferential cytotoxicity of PCA under nutrient-deprived conditions**. Human pancreatic cancer cells were incubated with PCA for 24h in NDM or DMEM.

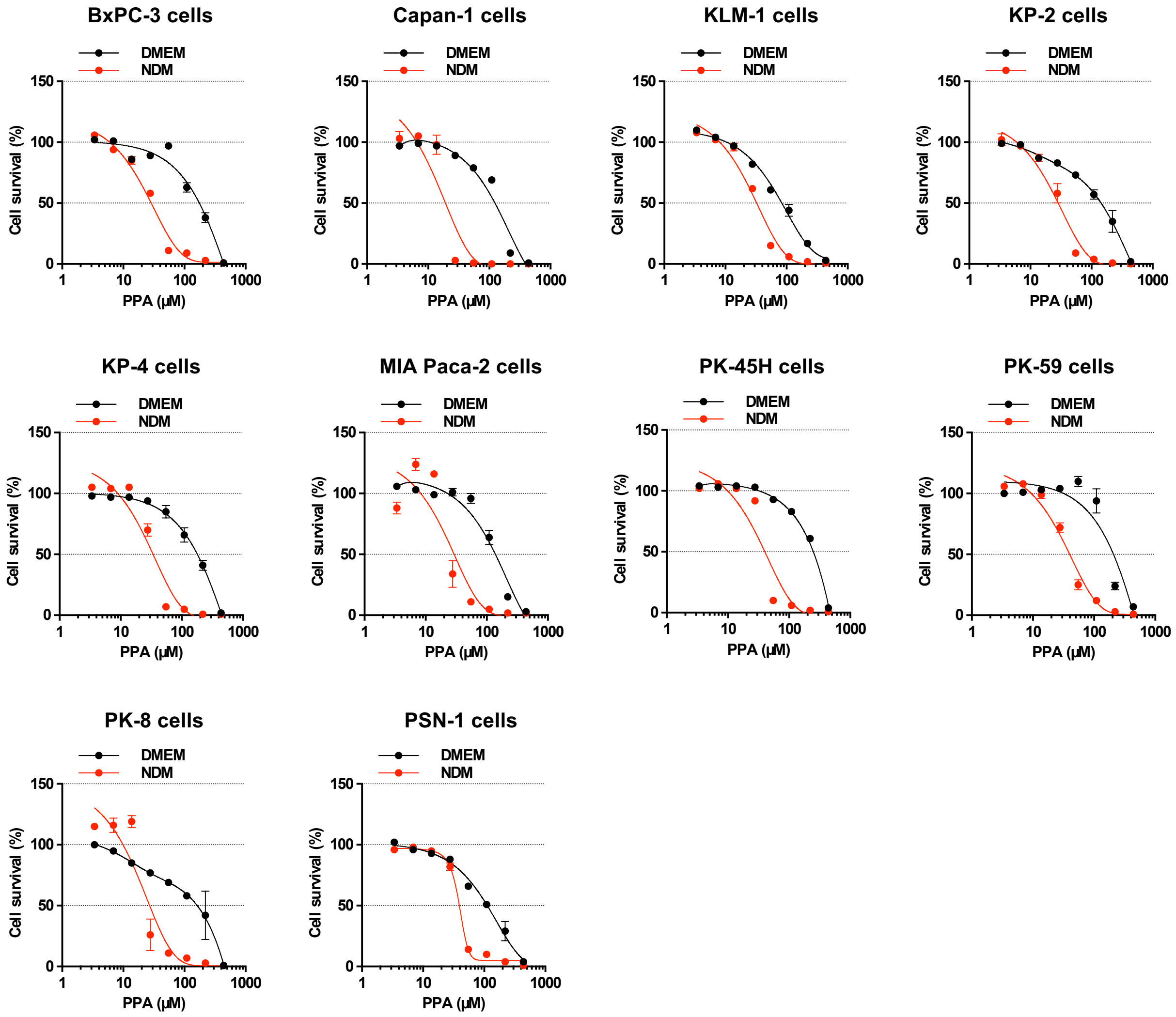

SUPPLEMENTAL FIGURE S1B. **Preferential cytotoxicity of PPA under nutrient-deprived conditions**. Human pancreatic cancer cells were incubated with PPA for 24h in NDM or DMEM.

Number of colonies		
	PCA	
	-	+
DMEM	143±15	135±5
NDM	105±20	18±2

Number of colonies		
	PPA	
	-	+
DMEM	148±15	141±9
NDM	115±4	24±6

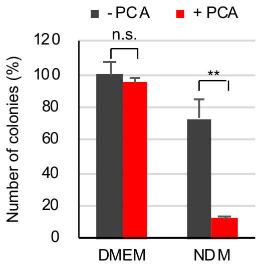

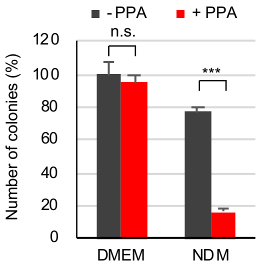

SUPPLEMENTAL FIGURE S1C. **Effect of PCA and PPA on colony formation of PANC-1 cells**. PANC-1 cells were incubated for 10 days in DMEM after treatment with 6 μM PCA or 4 μM PPA for 24 h in NDM or DMEM. Data are presented as mean ± S.D. of three independent experiments. *p* values were determined by two-tailed student's *t* test (**, $p < 0.01$; ***, $p < 0.001$; n.s., not significant).

	Cell line	Log GI_{50}
Br	*	
	HBC-4	-4.57
	BSY-1	-4.74
	HBC-5	-4.80
	MCF-7	-4.76
	MDA-MB-231	-4.60
CNS	*	
	U251	-4.47
	SF-268	-4.53
	SF-295	-4.48
	SF-539	-4.70
	SNB-75	-4.57
	SNB-78	-4.50
Co	*	
	HCC2998	-4.52
	KM-12	-4.48
	HT-29	-4.47
	HCT-15	-4.55
	HCT-116	-4.50
Lu	*	
	NCI-H23	-4.62
	NCI-H226	-4.57
	NCI-H522	-4.91
	NCI-H460	-4.54
	A549	-4.51
	DMS273	-4.77
	DMS114	-4.60
Me	*	
	LOX-IMVI	-4.64
Ov	*	
	OVCAR-3	-4.70
	OVCAR-4	-4.59
	OVCAR-5	-4.62
	OVCAR-8	-4.54
	SK-OV-3	-4.57
Re	*	
	RXF-631L	-4.60
	ACHN	-4.55
St	*	
	St-4	-4.45
	MKN1	-4.59
	MKN7	-4.49
	MKN28	-4.46
	MKN45	-4.38
	MKN74	-4.53
xPg	*	
	DU-145	-4.47
	PC-3	-4.46
	*	
MG-MID		-4.57
Delta		0.34
Range		0.53

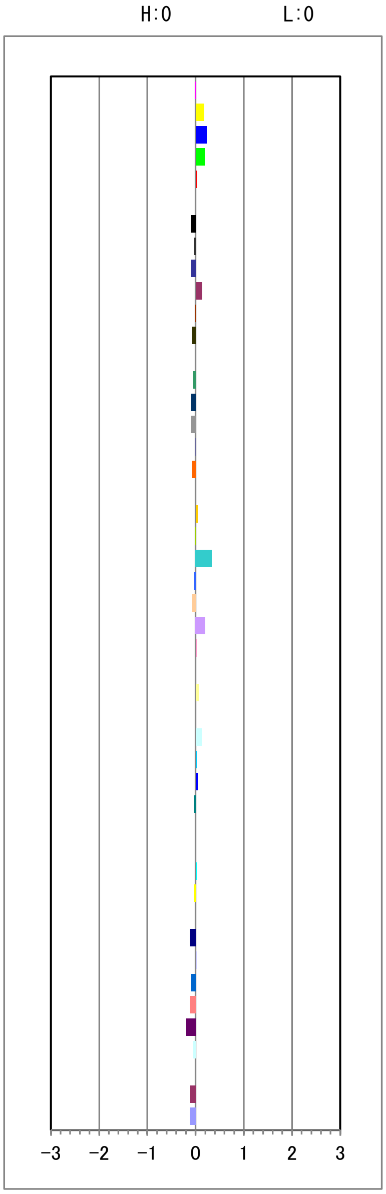

SUPPLEMENTAL FIGURE S2A**. Growth inhibition of PCA against a panel of 39 human cancer cell lines.** The X- axis shows the difference in logarithmic scale between the mean of Log GI50 value for all 39 cell lines (MG-MID, expressed as zero) and the Log GI_{50} for each cell line in the JFCR39 panel. Columns to the right of zero indicate the sensitivity of the cell lines to PCA and columns to the left indicate the resistance. MG-MID=mean of Log GI_{50} values for all 39 cell lines; delta = difference between the MG-MID and Log GI_{50} value for the most sensitive cell line; range = reference between the Log GI50 value for the most resistant cell line and the most sensitive cell line.

Br	*	
	HBC-4	-4.44
	BSY-1	-4.48
	HBC-5	-4.39
	MCF-7	-4.43
	MDA-MB-231	-4.29
CNS	*	
	U251	-4.29
	SF-268	-4.43
	SF-295	-4.18
	SF-539	-4.61
	SNB-75	-4.36
	SNB-78	-4.27
Co	*	
	HCC2998	-4.39
	KM-12	-4.34
	HT-29	-4.00
	HCT-15	-4.07
	HCT-116	-4.24
Lu	*	
	NCI-H23	-4.42
	NCI-H226	-4.31
	NCI-H522	-4.68
	NCI-H460	-4.06
	A549	-4.00
	DMS273	-4.26
	DMS114	-4.41
Me	*	
	LOX-IMVI	-4.52
Ov	*	
	OVCAR-3	-4.44
	OVCAR-4	-4.00
	OVCAR-5	-4.25
	OVCAR-8	-4.33
	SK-OV-3	-4.00
Re	*	
	RXF-631L	-4.00
	ACHN	-4.04
St	*	
	St-4	-4.38
	MKN1	-4.41
	MKN7	-4.00
	MKN28	-4.00
	MKN45	-4.16
	MKN74	-4.18
xPg	*	
	DU-145	-4.00
	PC-3	-4.09
	*	
MG-MID		-4.26
Delta		0.42
Range		0.68

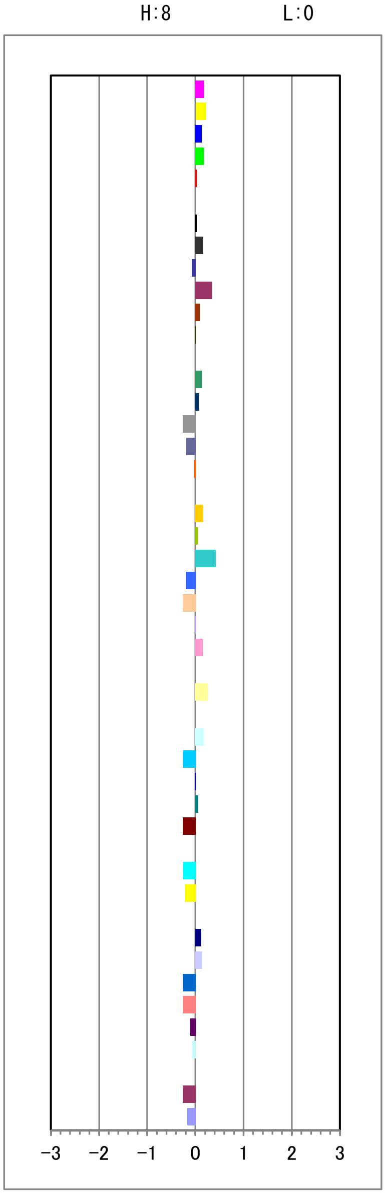

SUPPLEMENTAL FIGURE S2B**. Growth inhibition of PPA against a panel of 39 human cancer cell lines.**

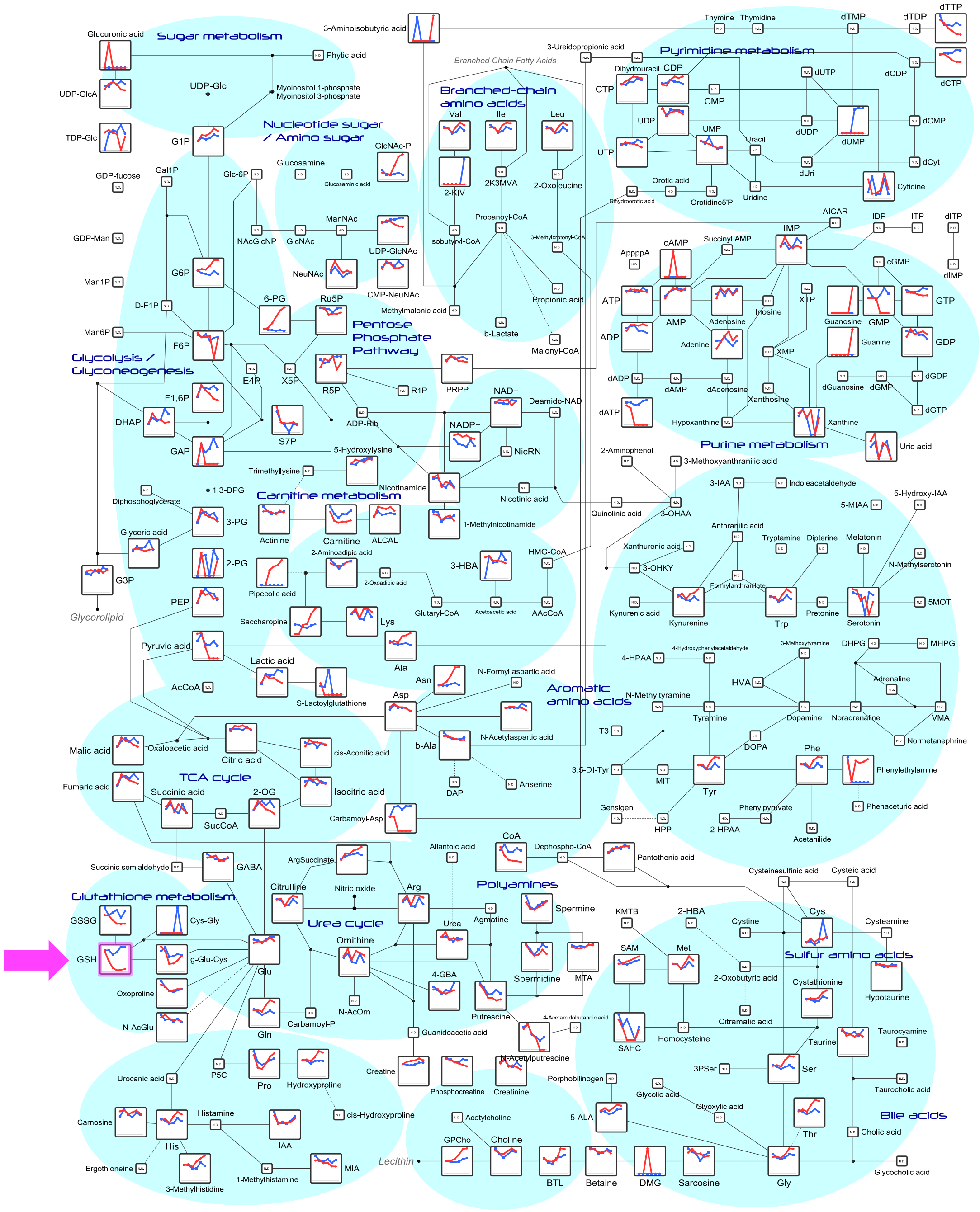

SUPPLEMENTAL FIGURE S2C. **Effect of PCA on intracellular concentration of metabolites involved in central metabolic pathways.**

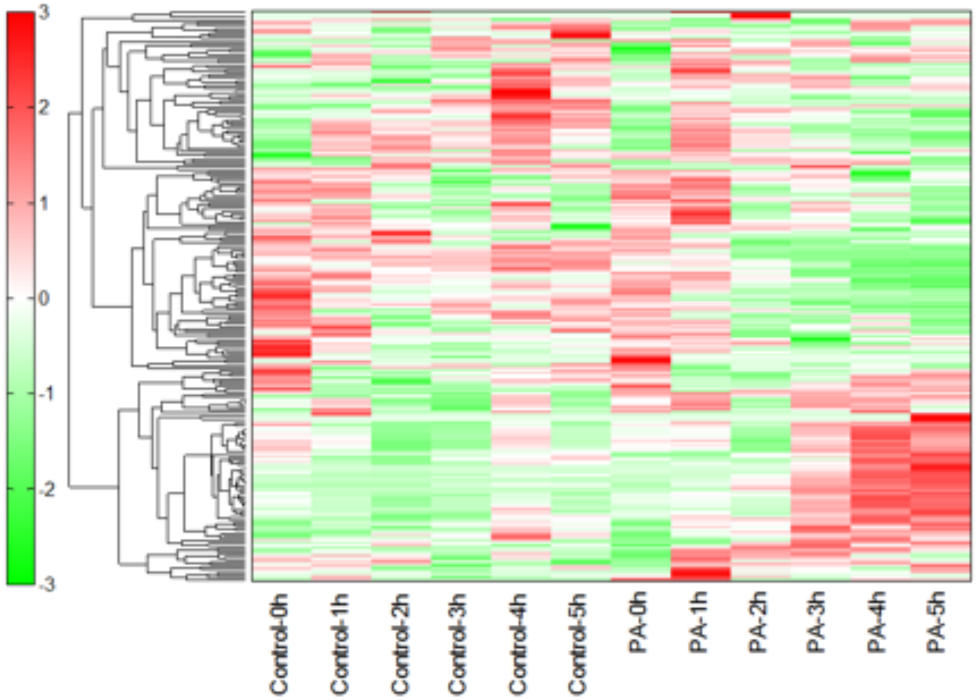

SUPPLEMENTAL FIGURE S2D. **Heat map with hierarchical clustering of metabolite levels involved in central metabolic pathways.** Each metabolite was normalized by dividing by the median of all samples. Data colored in the red-white-green scheme indicate a relatively higher, average, and lower concentration, respectively. Hierarchical cluster analysis was performed using PeakStat ver.3.18 software (HMT). Data is listed in Supplemental Table S1.

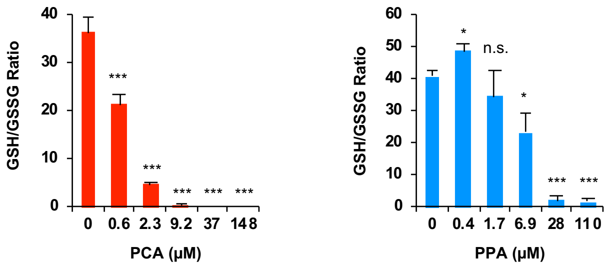

SUPPLEMENTAL FIGURE S2E. **Effect of PCA and PPA concentrations on the GSH/GSSG ratio in PANC-1 cells.** Data are presented as mean ± S.D. of three independent experiments. *p* values were determined by two-tailed student's *t* test (*, $p < 0.05$; ***, $p < 0.001$; n.s., not significant).

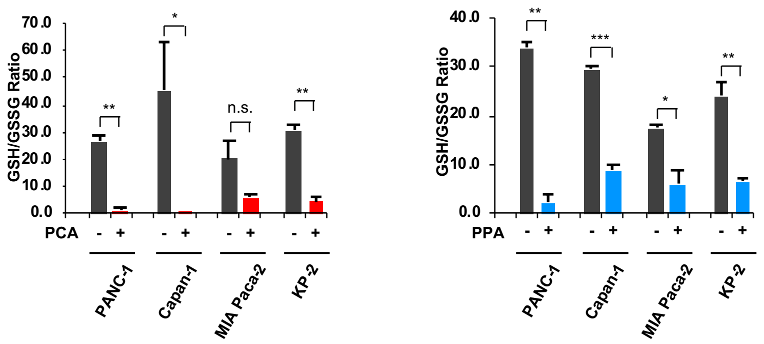

SUPPLEMENTAL FIGURE S2F. **Effect of PCA and PPA on the GSH/GSSG ratio in human pancreatic cancer cells.** Data are presented as mean ± S.D. of three independent experiments. *p* values were determined by two-tailed student's *t* test (*, $p < 0.05$; **, $p < 0.01$; ***, $p < 0.001$; n.s., not significant).

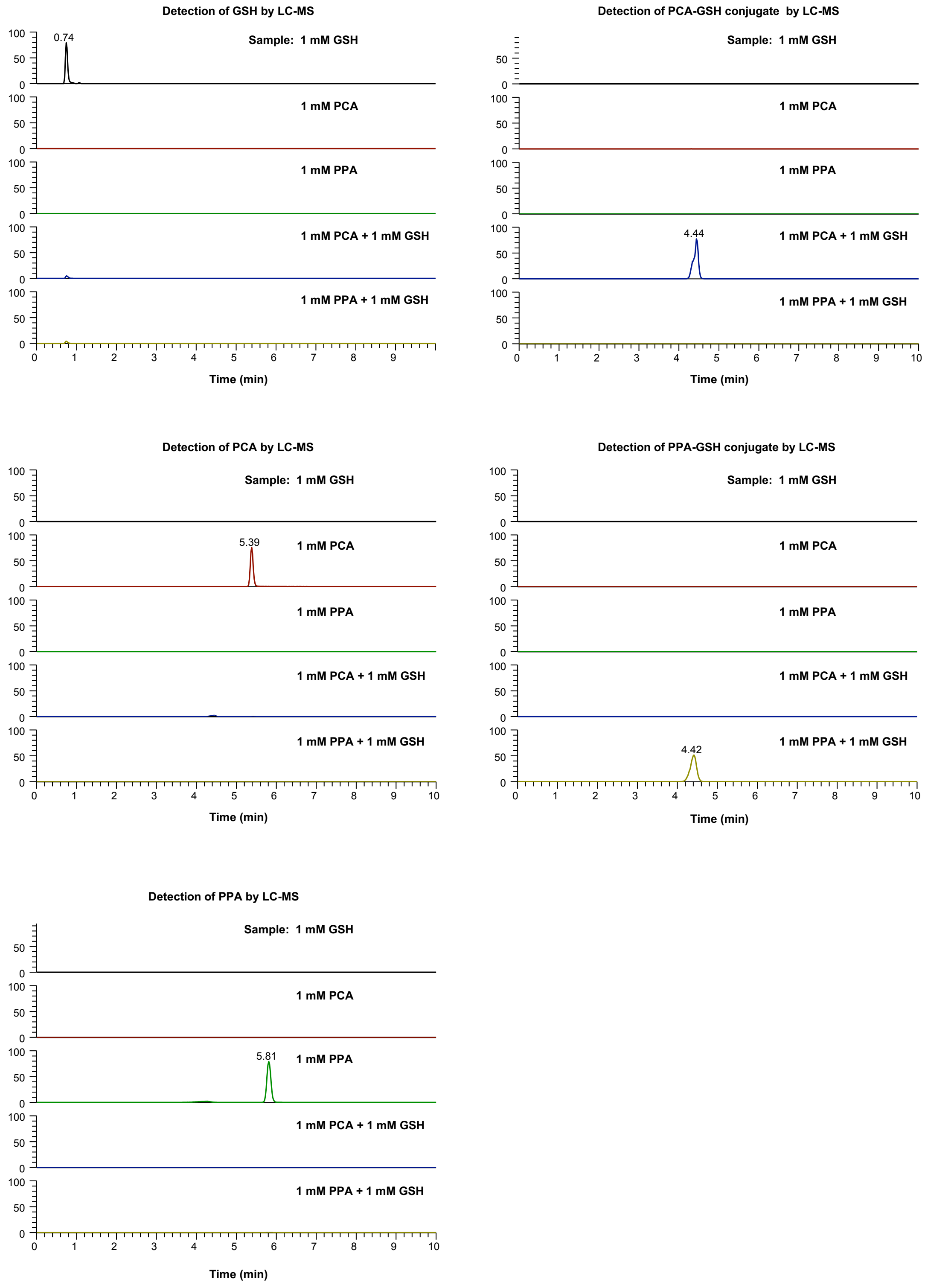

SUPPLEMENTAL FIGURE S2G. **PCA and PPA directly bind to GSH. PCA/PPA-GSH conjugates were detected by LC-MS.**

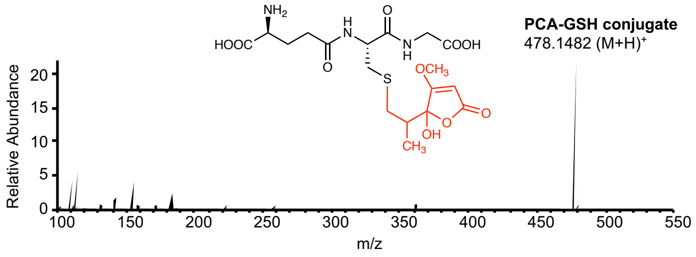

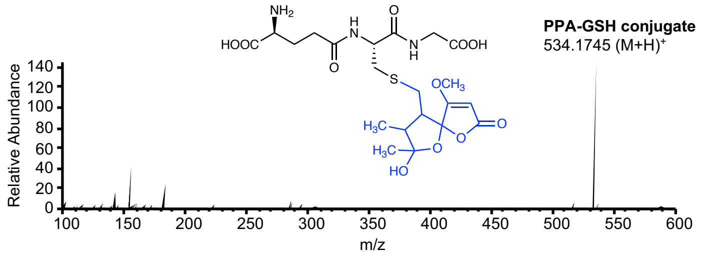

SUPPLEMENTAL FIGURE S2H. **Mass spectra and proposed chemical structures of PCA-GSH and PPA-GSH conjugates.**

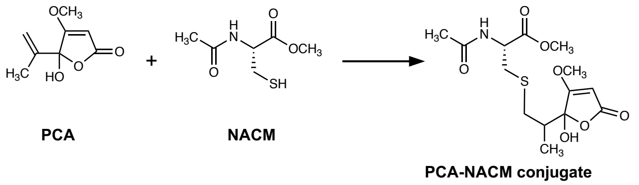

SUPPLEMENTAL FIGURE S2I. **PCA binds to NACM, forming the PCA–NACM conjugate**

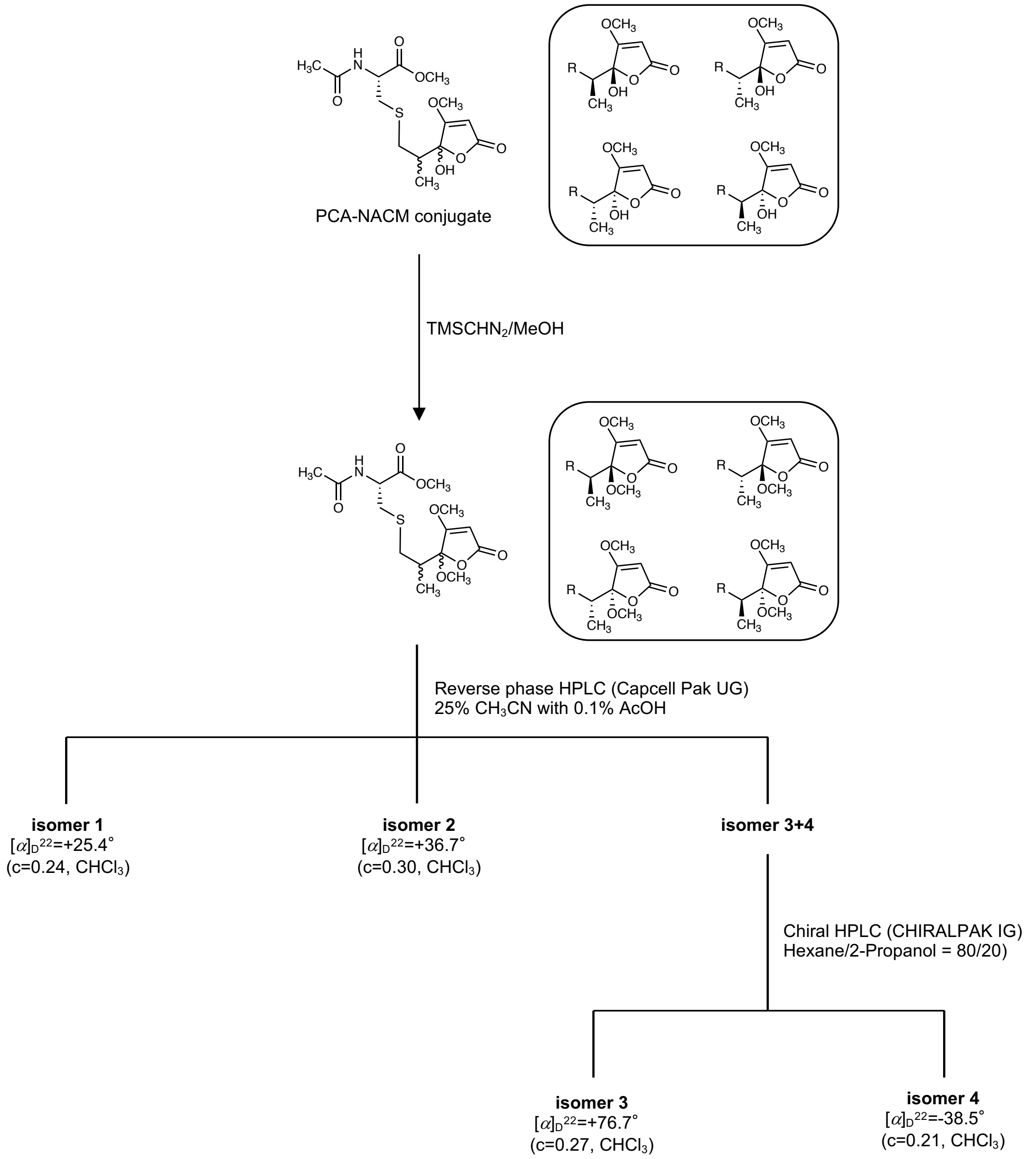

SUPPLEMENTAL FIGURE S2J. **Isolation procedure of PCA-NACM conjugate derivatives.**

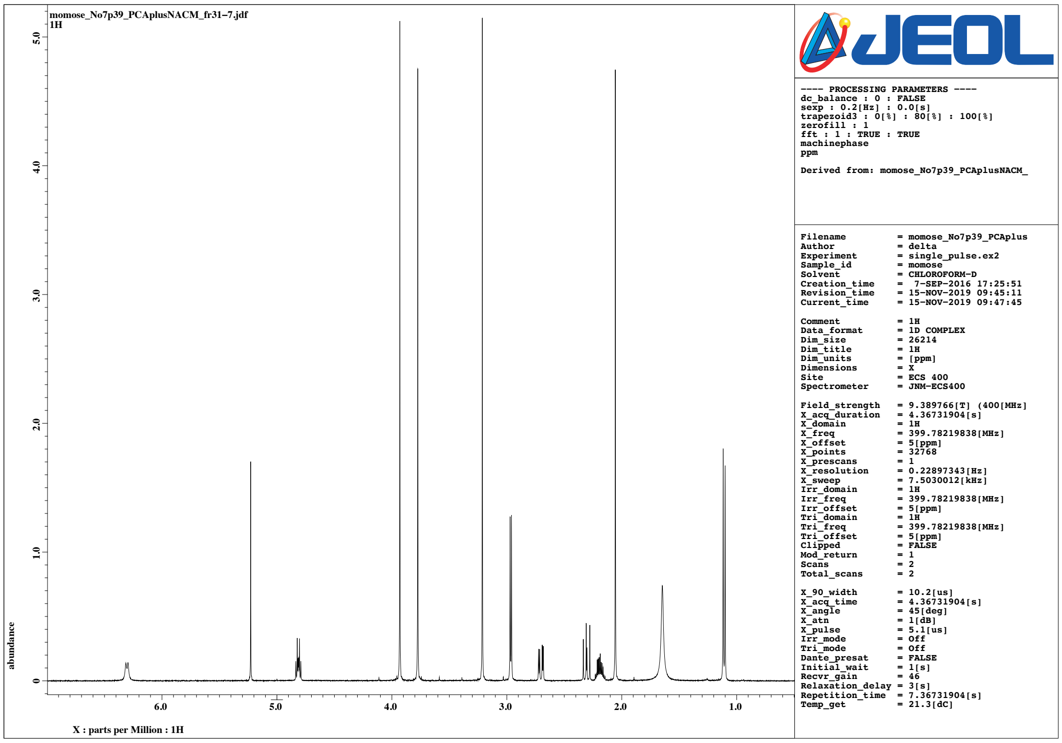

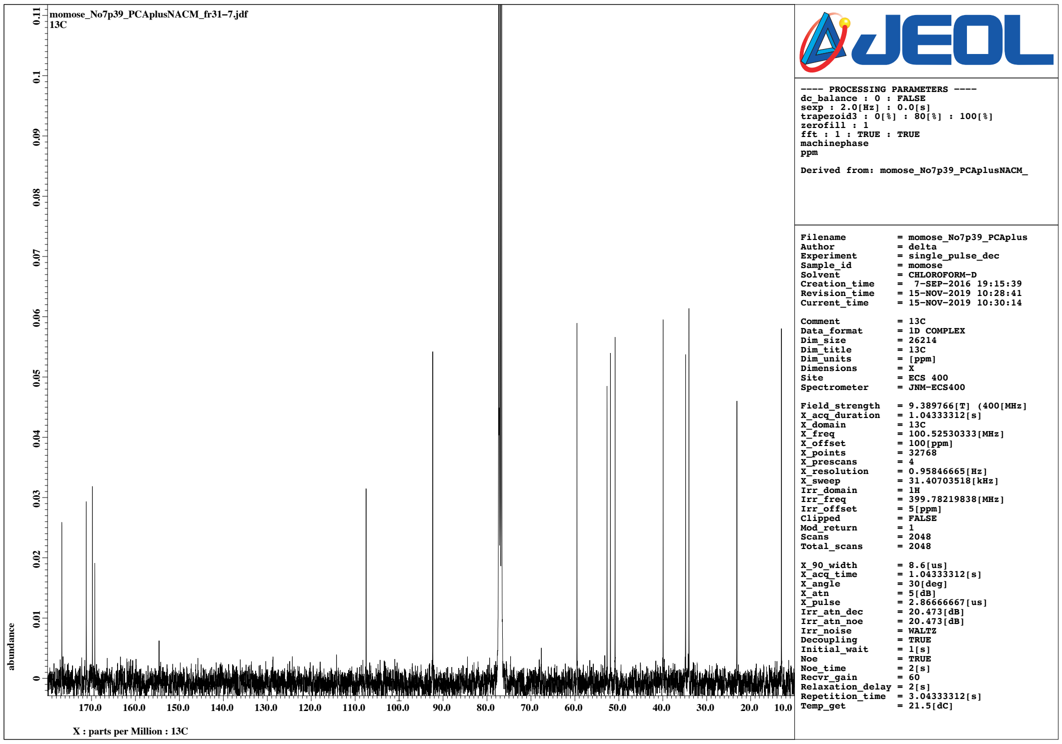

SUPPLEMENTAL FIGURE S2K. **^{1}H and ^{13}C NMR spectrum of isomer 1 in CDCl$_3$.**

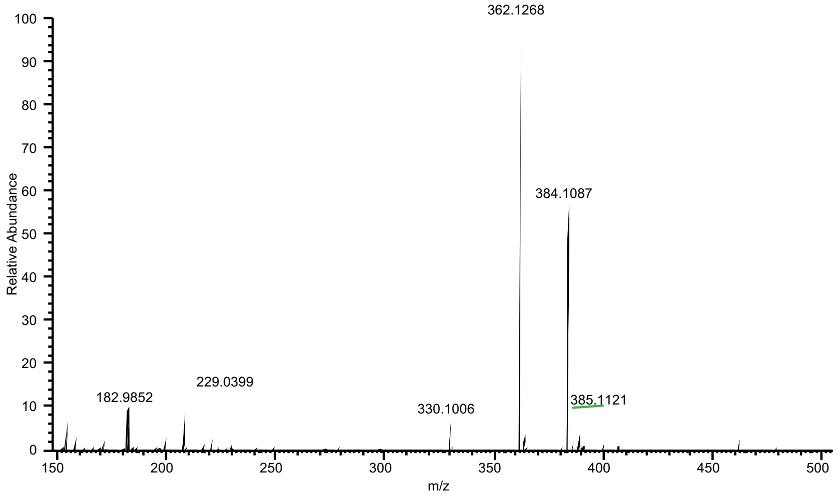

SUPPLEMENTAL FIGURE S2L. **MS spectrum of isomer 1 obtained in the positive mode.**

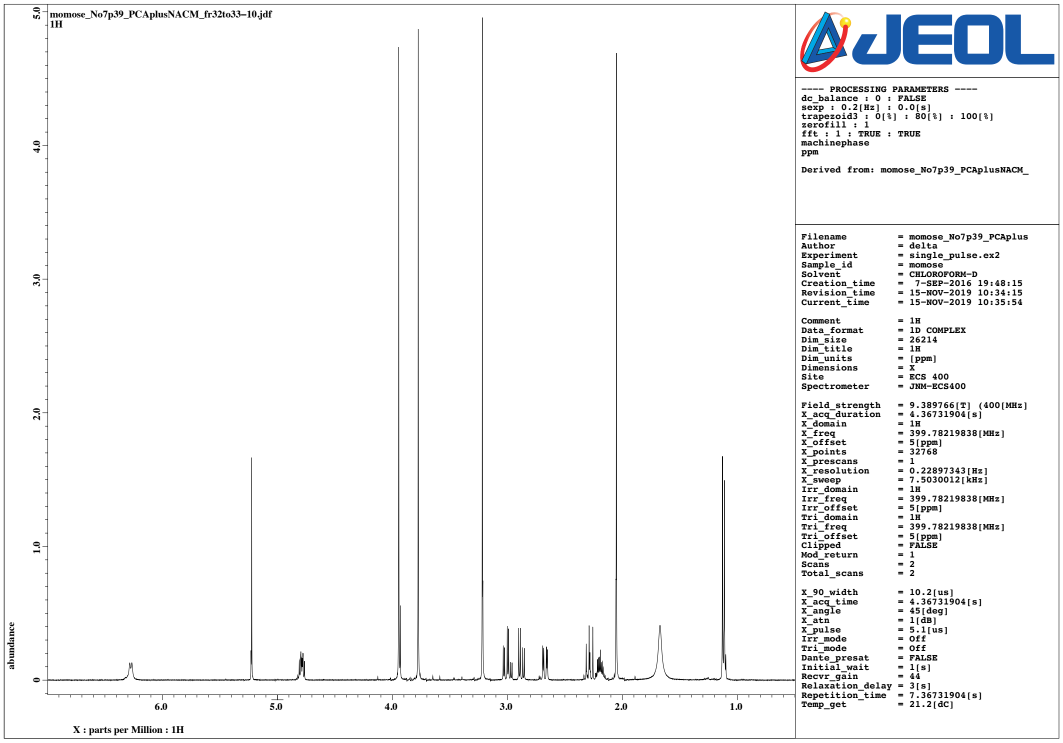

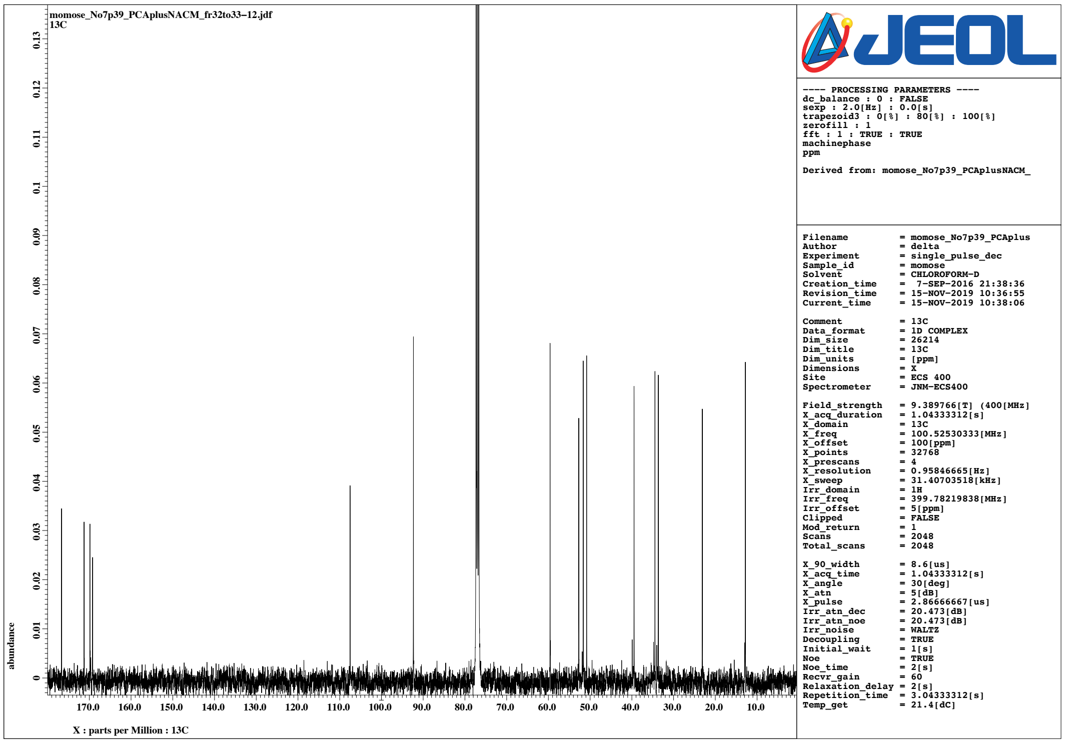

SUPPLEMENTAL FIGURE S2M. **^{1}H and ^{13}C NMR spectrum of isomer 2 in $CDCl_3$.**

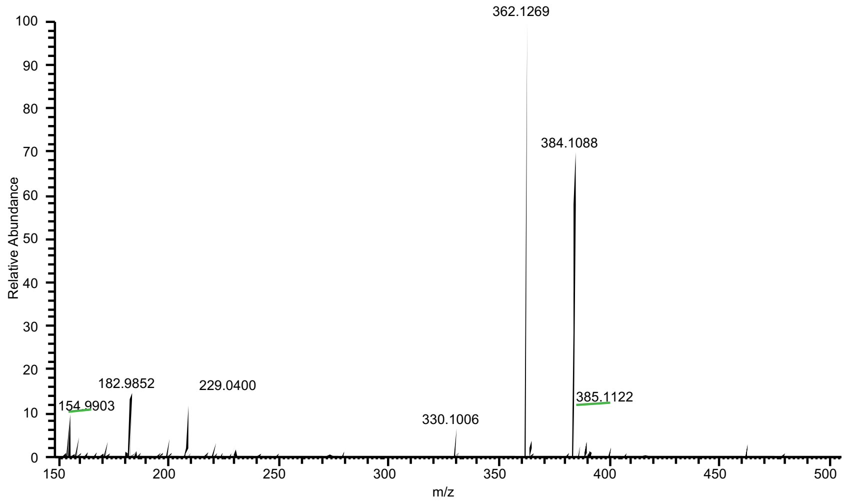

SUPPLEMENTAL FIGURE S2N. **MS spectrum of isomer 2 obtained in the positive mode.**

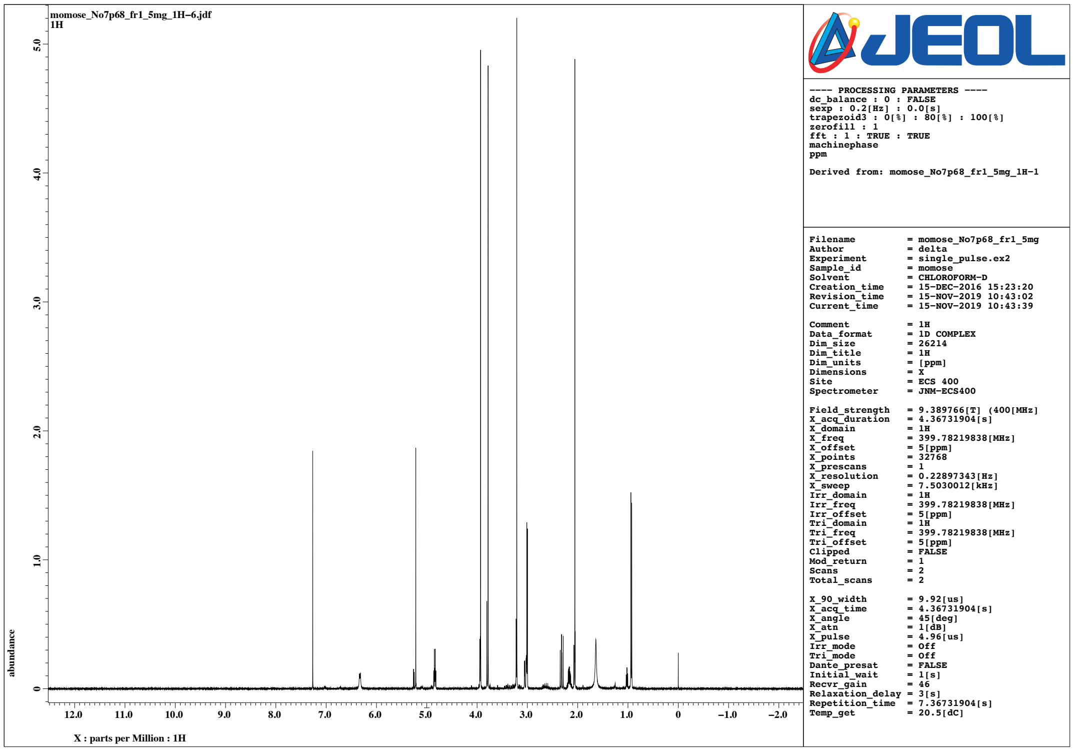

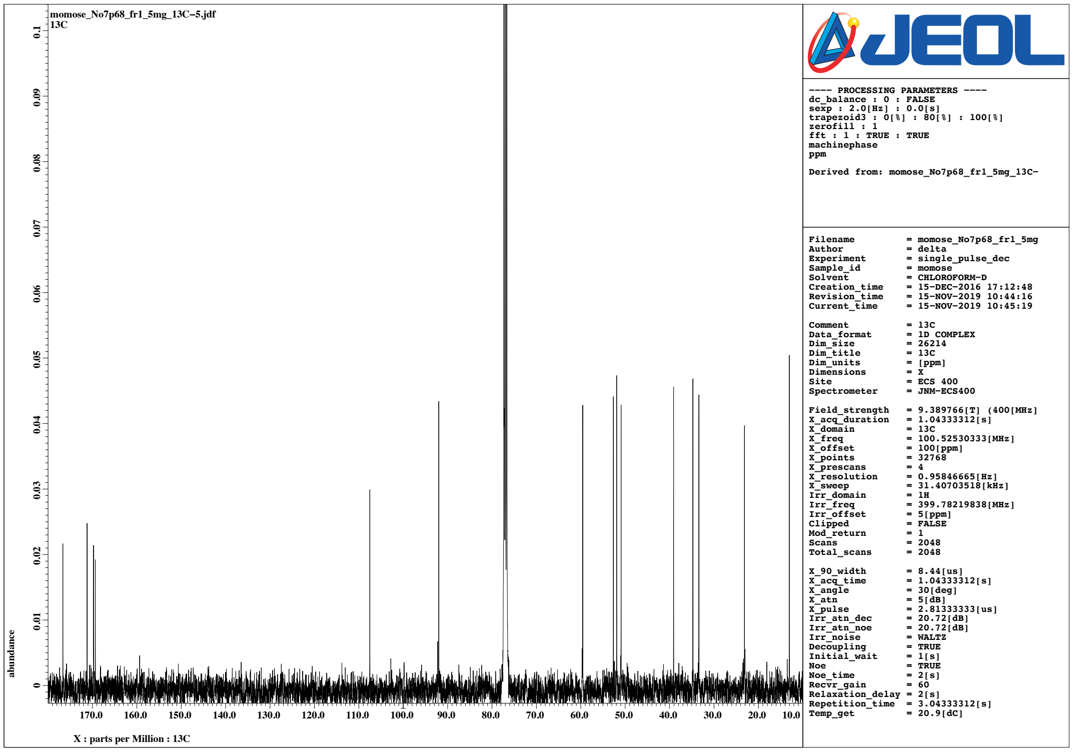

SUPPLEMENTAL FIGURE S2O. **^{1}H and ^{13}C NMR spectrum of isomer 3 in $CDCl_3$.**

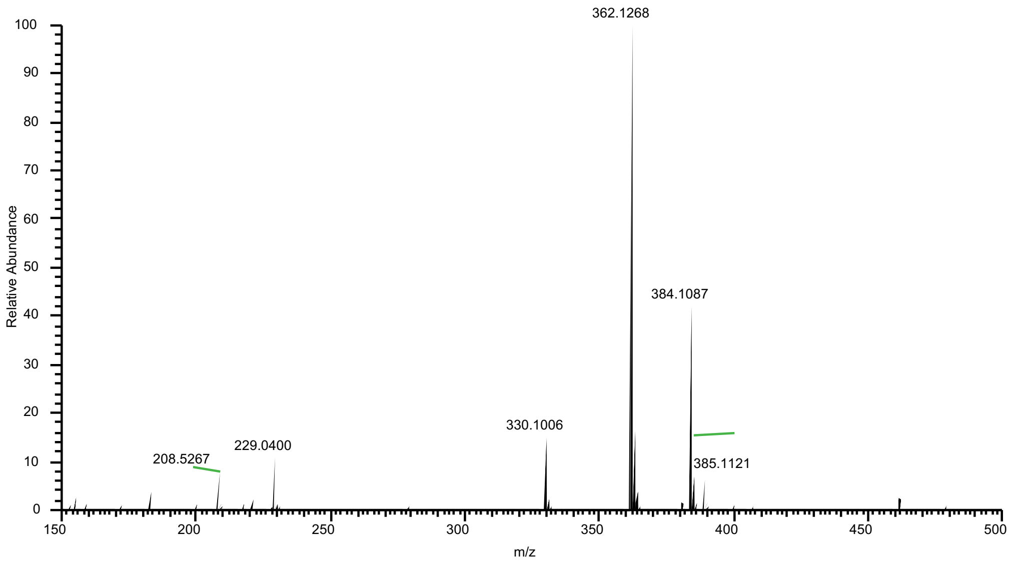

SUPPLEMENTAL FIGURE S2P. **MS spectrum of isomer 3 obtained in the positive mode.**

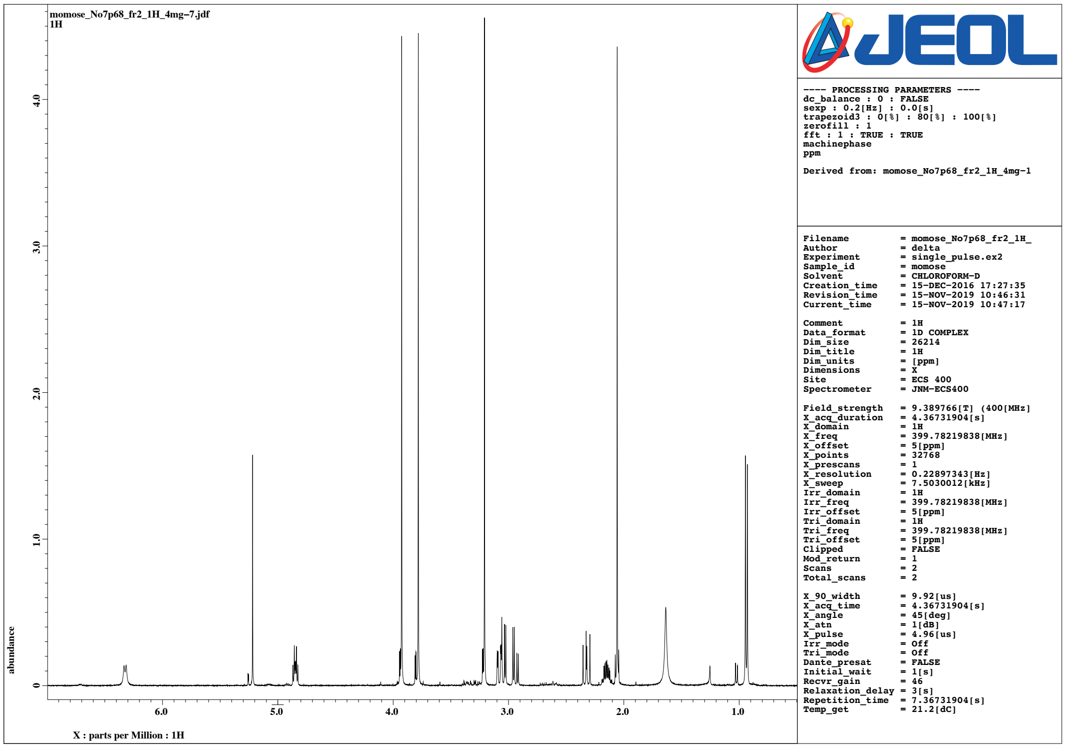

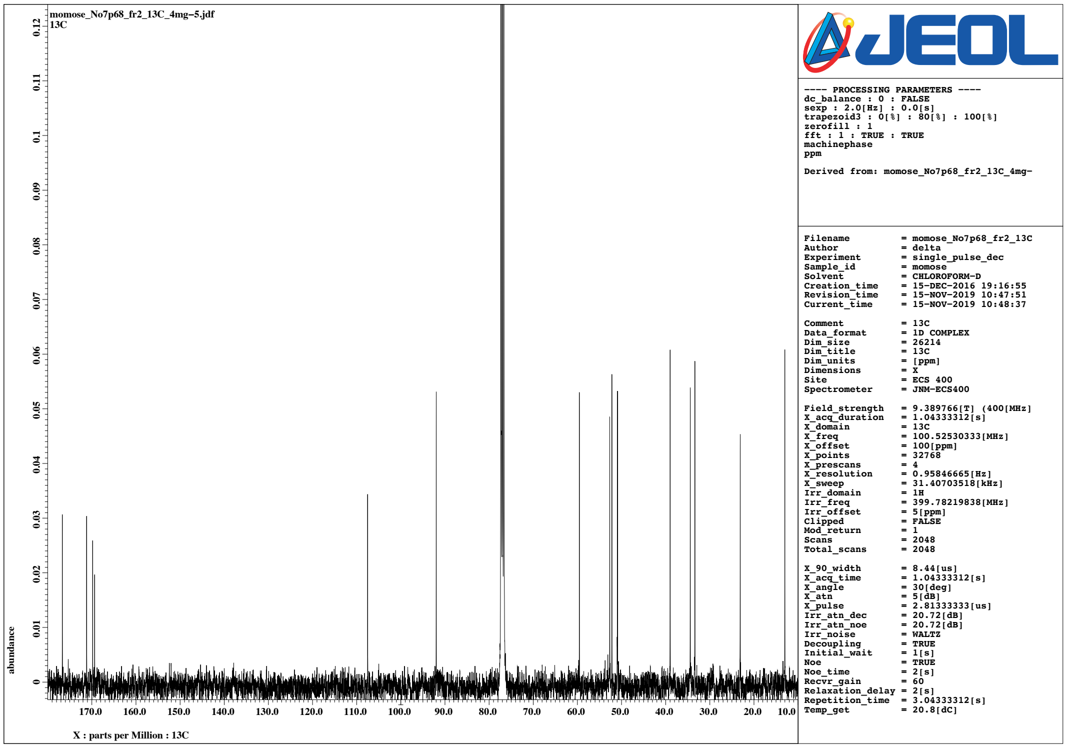

SUPPLEMENTAL FIGURE S2Q. **^{1}H and ^{13}C NMR spectrum of isomer 4 in $CDCl_3$.**

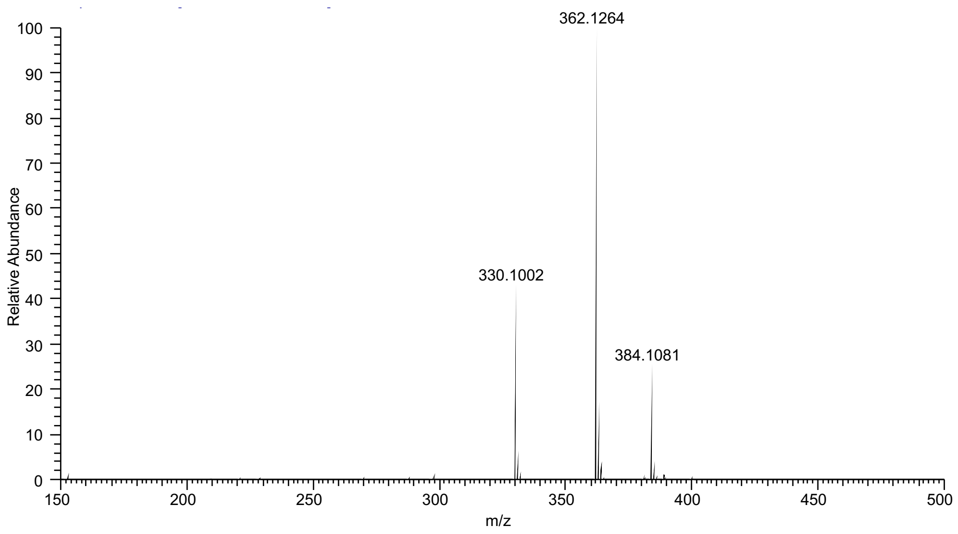

SUPPLEMENTAL FIGURE S2R. **MS spectrum of isomer 4 obtained in the positive mode.**

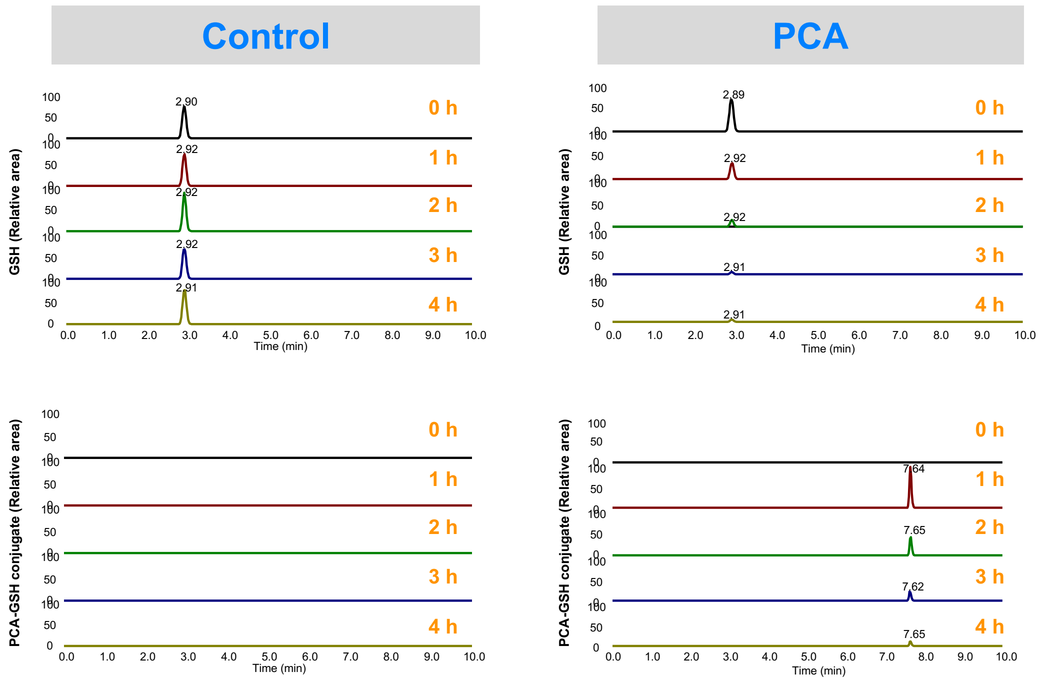

SUPPLEMENTAL FIGURE S2S. **PCA decreases GSH and forms the PCA–Cys conjugate in PANC-1 cells.** PANC-1 cells were treated with 59 µM PCA for the indicated times. Metabolites extracted from PANC-1 cells were determined by LC-MS.

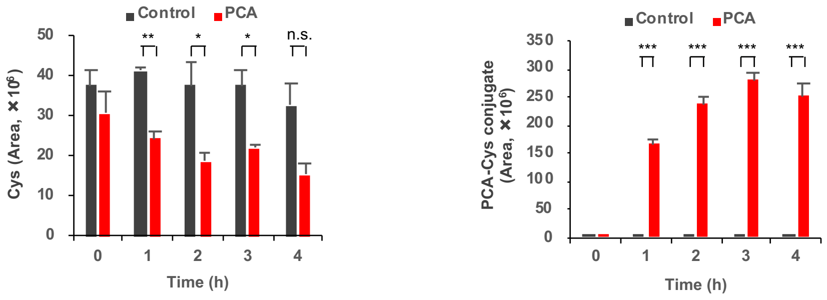

SUPPLEMENTAL FIGURE S2T. **Formation of the PCA–Cys conjugate in BxPC-3 cells.** Intracellular metabolites of BxPC-3 cells treated with 59 μM PCA were measured by LC-MS. Cys and PCA–Cys conjugate were monitored at *m/z* 122.0270±0.0006 and *m/z* 292.0849±0.0015, respectively. Data are presented as mean ± S.D. of three independent experiments. *p* values were determined by two-tailed student's *t* test (*, $p < 0.05$; **, $p < 0.01$; ***, $p < 0.001$; n.s., not significant).

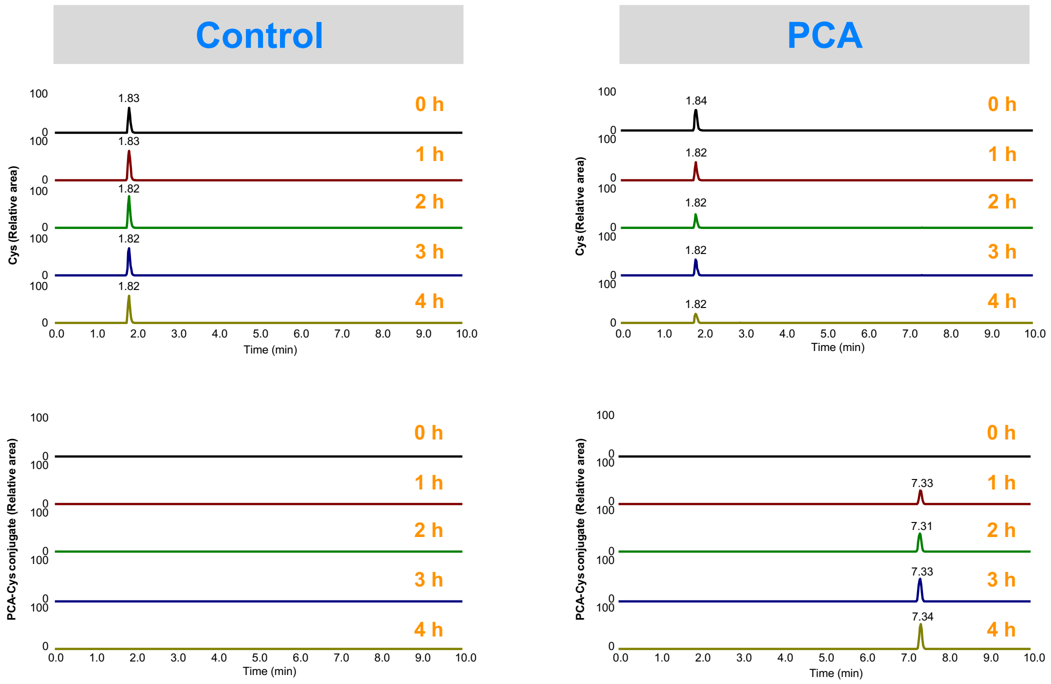

SUPPLEMENTAL FIGURE S2U. **PCA decreases Cys and forms the PCA–Cys conjugate in BxPC-3 cells.** BxPC-3 cells were treated with 59 μM PCA for the indicated times. Metabolites extracted from BxPC-3 cells were determined by LC-MS.

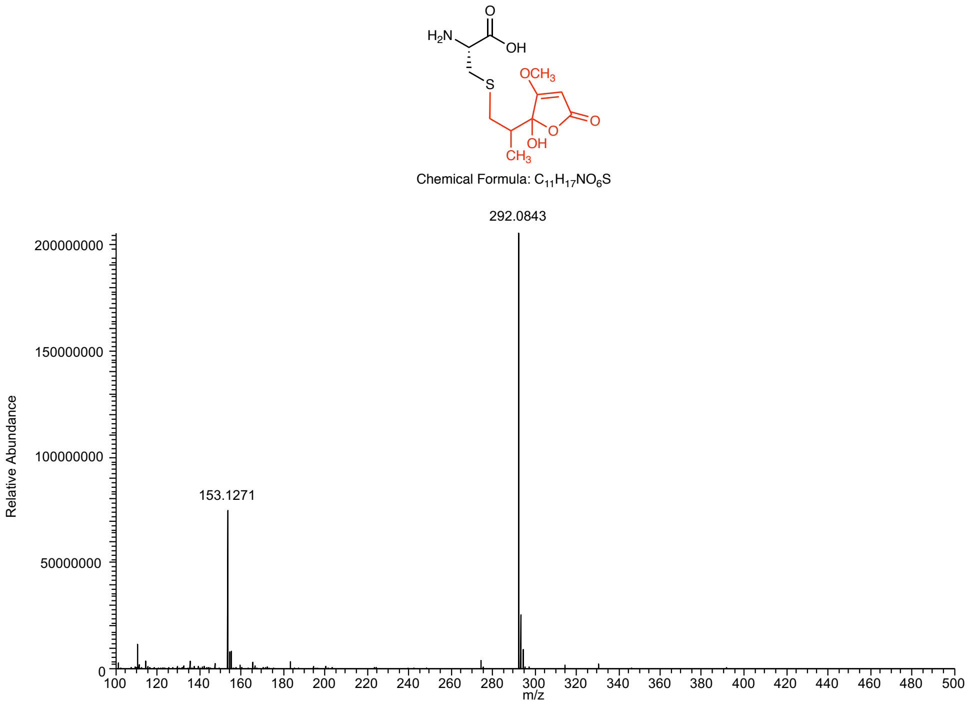

SUPPLEMENTAL FIGURE S2V. **MS spectrum of the PCA–Cys conjugate obtained in the positive mode.**

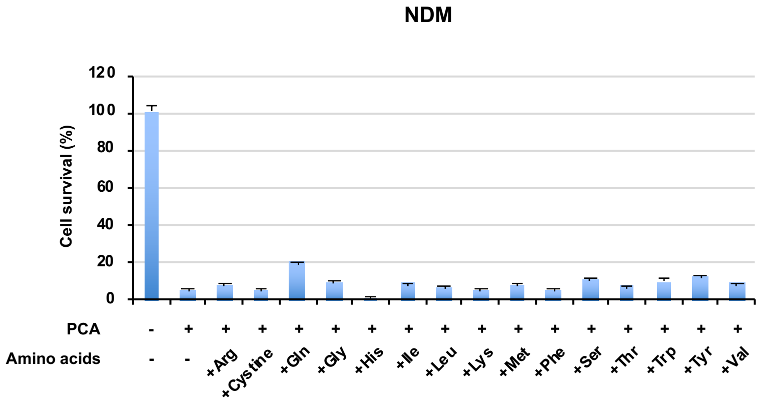

SUPPLEMENTAL FIGURE S3A. **Effect of 15 amino acids on cytotoxicity of PCA under nutrient-deprived conditions.** PANC-1 cells were incubated with 118 μM PCA for 24 h in NDM with individual amino acids.

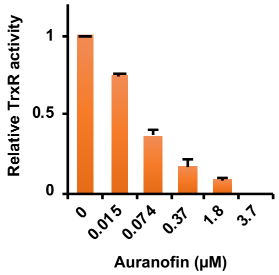

SUPPLEMENTAL FIGURE S4A. **Auranofin inhibited TrxR activity in PSN-1 cells**

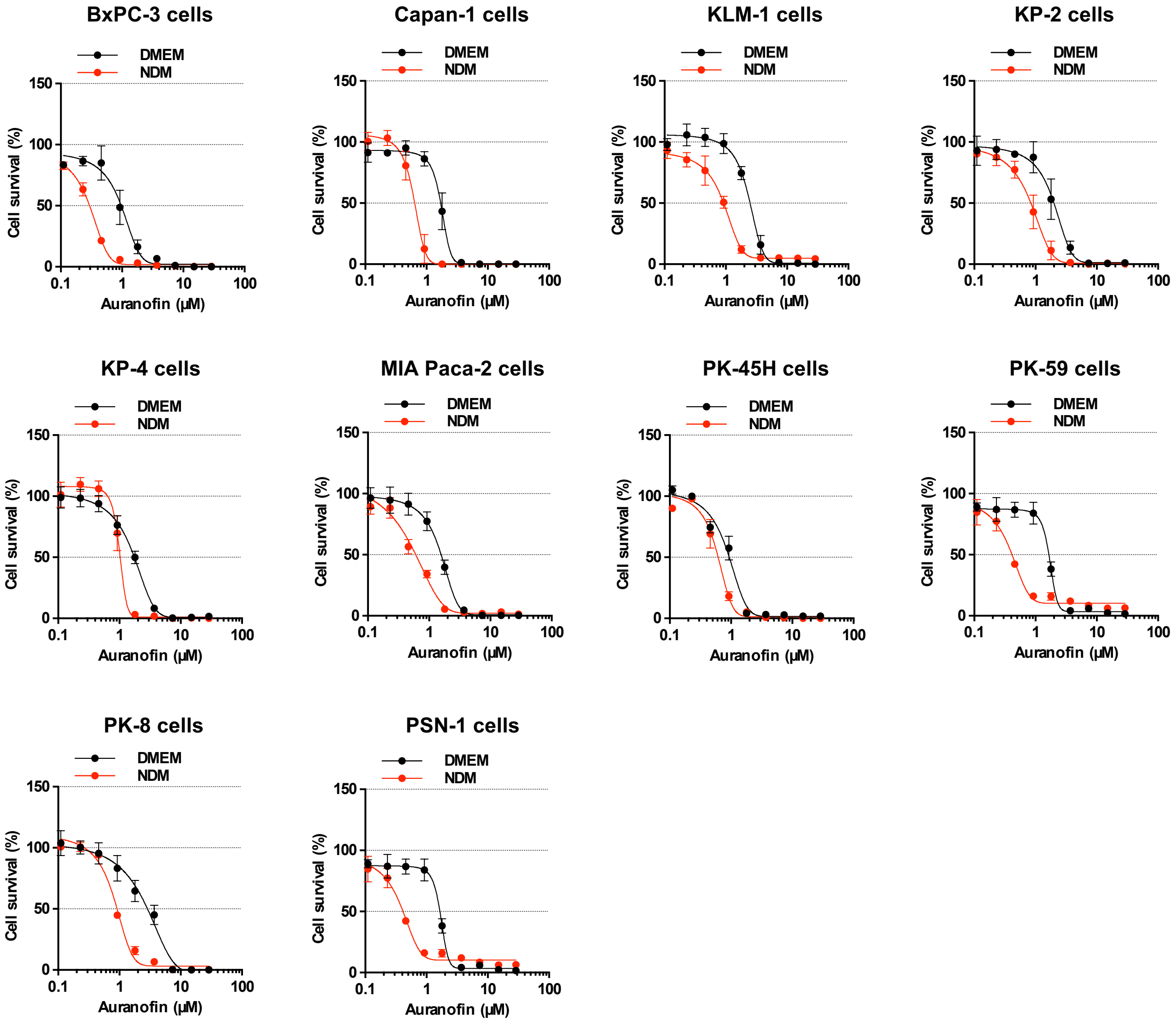

SUPPLEMENTAL FIGURE S4B. **Preferential cytotoxicity of auranofin under nutrient-deprived conditions.** Human pancreatic cancer cells were incubated with auranofin for 24 h in NDM or DMEM.

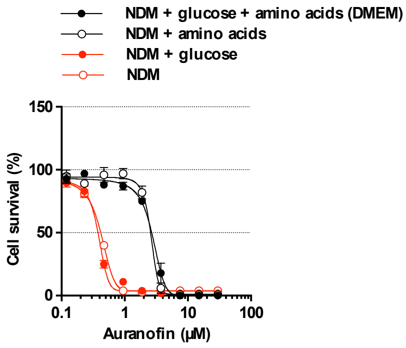

SUPPLEMENTAL FIGURE S4C. **Effect of glucose and amino acids on preferential cytotoxicity of auranofin under nutrient-deprived conditions.** PANC-1 cells were incubated with auranofin for 24 h in NDM to which glucose or amino acids were added.

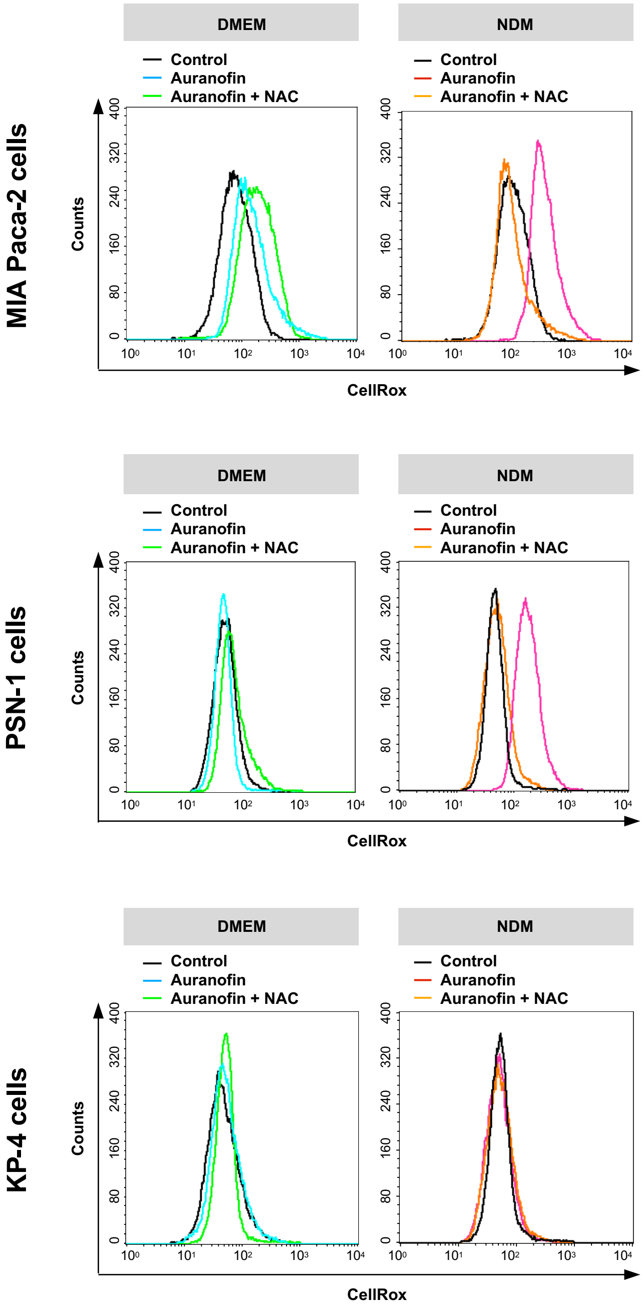

SUPPLEMENTAL FIGURE S4D. **Intracellular ROS levels.** MIA Paca-2, PSN-1 and KP-4 cells were treated with 1.5 µM auranofin for 12 h in the absence or presence of 5 mM NAC.

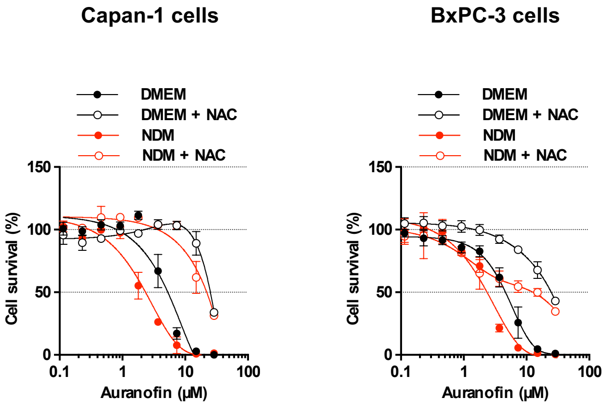

SUPPLEMENTAL FIGURE S4E. **NAC abrogated the suppression of cell growth by auranofin.** Capan-1 and BxPC-3 cells were incubated with auranofin for 24 h in the absence or presence of 5 mM NAC.

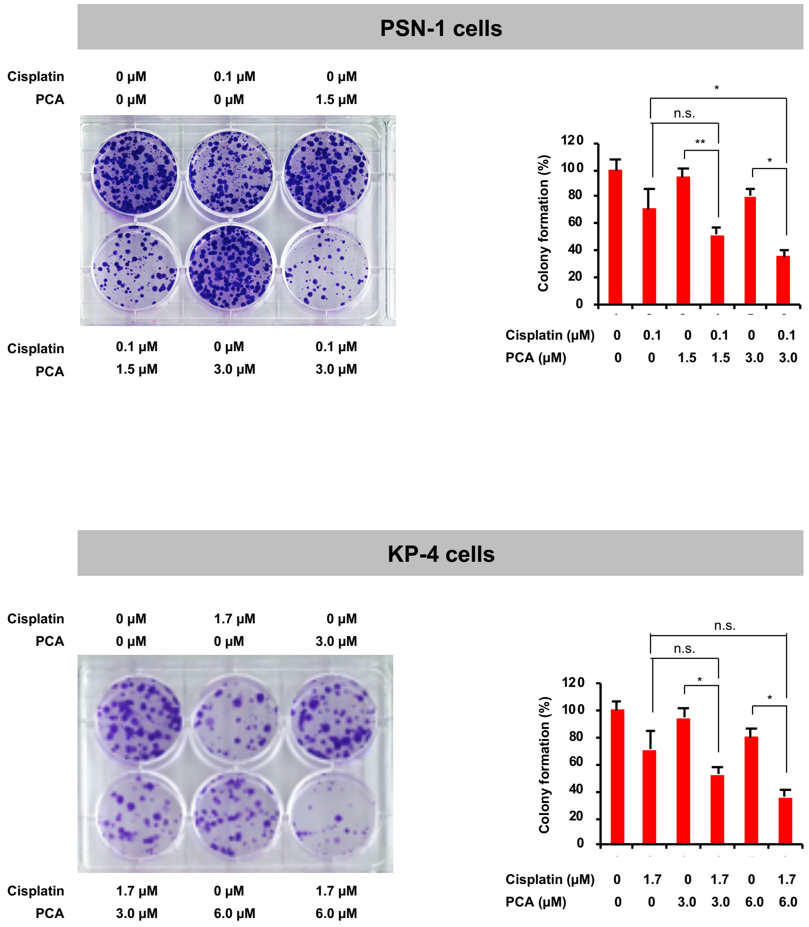

SUPPLEMENTAL FIGURE S4F. **The combined effect of cisplatin and PCA on colony formation of PSN-1 and KP-4 cells.** The cells were incubated with PCA and/or cisplatin for 14 days in DMEM. Data are presented as mean ± S.D. of three independent experiments. *p* values were determined by two-tailed student's *t* test (*, $p < 0.05$; **, $p < 0.01$; n.s., not significant).

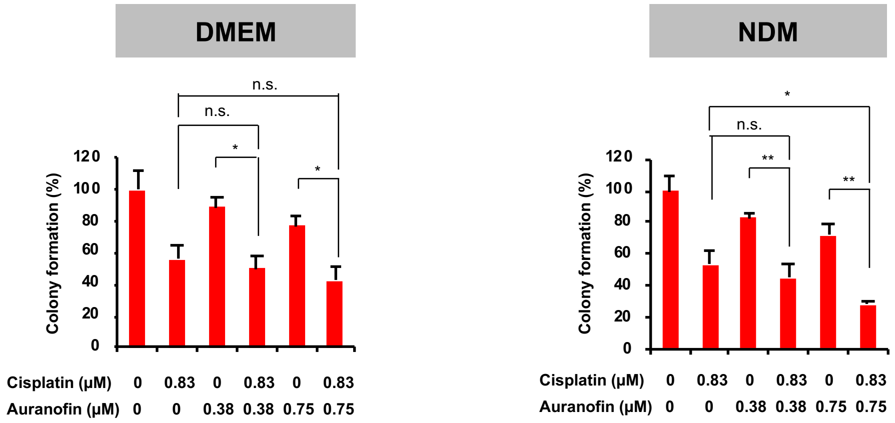

SUPPLEMENTAL FIGURE S4G **The combined effect of cisplatin and auranofin on colony formation of PANC-1 cells.** The cells were incubated for 14 days in DMEM after treatment with cisplatin and/or auranofin for 24 h in DMEM or NDM. Data are presented as mean ± S.D. of three independent experiments. *p* values were determined by two-tailed student's *t* test (*, $p < 0.05$; **, $p < 0.01$; n.s., not significant).

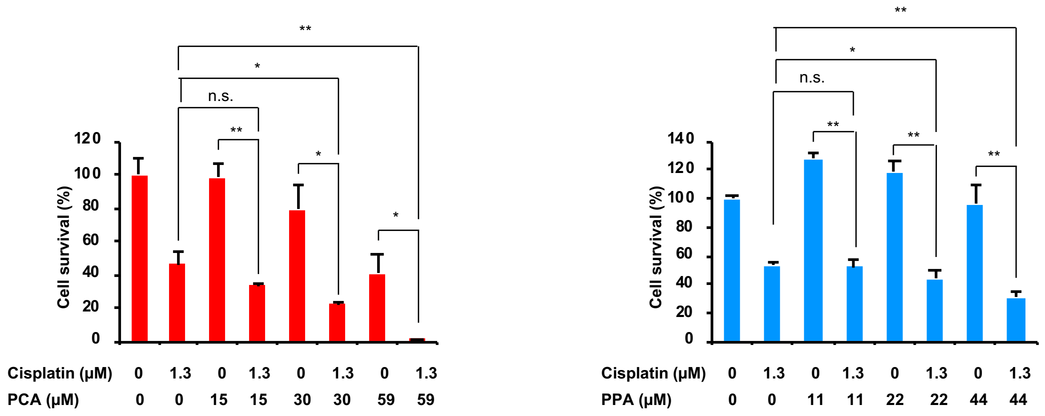

SUPPLEMENTAL FIGURE S4H. **The combined effect of cisplatin and PCA/PPA on 3D spheroid models of PSN-1 cells.** PSN-1 cells were seeded in 96-well spheroid cell culture plates (MS-9096U, Sumitomo Bakelite, Tokyo, Japan) at a density of 5 × 10^3 cells/well and incubated for 3 days. Cisplatin and/or PCA/PPA were added into each well, and the cells were incubated for 4 days. Cell viability was determined by the ATPlite assay system (PerkinElmer). Data are presented as mean ± S.D. of three independent experiments. p values were determined by two-tailed student's t test (*, $p < 0.05$; **, $p < 0.01$; n.s., not significant).

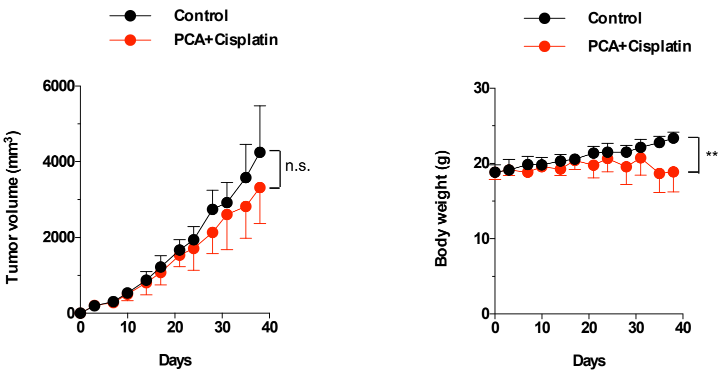

SUPPLEMENTAL FIGURE S4I. **The combined effect of cisplatin and PCA.** Cisplatin (2.5 mg/kg, i.v. n = 5) and PCA (50 mg/kg, i.p., n = 5) were administered once a week to PSN-1 tumor-bearing mice. p values were determined by two-tailed student's t test (**, $p < 0.01$; n.s., not significant).

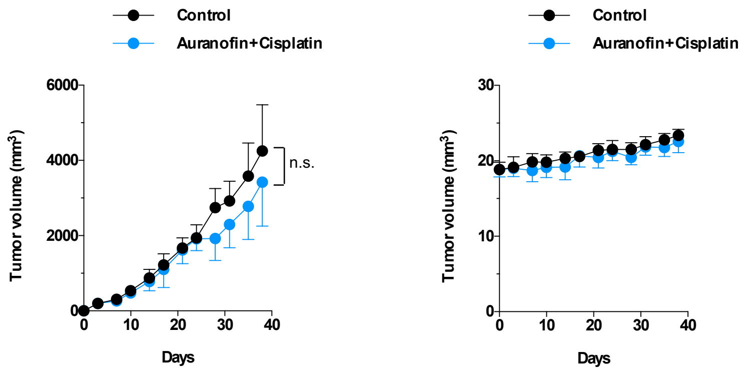

SUPPLEMENTAL FIGURE S4J. **The combined effect of cisplatin and auranofin.** Cisplatin (2.5 mg/kg, i.v., *n* = 5) and auranofin (12.5 mg/kg, i.p., *n* = 5) were administered once a week to PSN-1 tumor-bearing mice. *p* values were determined by two-tailed student's *t* test (n.s., not significant).
